# Personal experiences bridge moral and political divides better than facts

**DOI:** 10.1073/pnas.2008389118

**Published:** 2021-01-25

**Authors:** Emily Kubin, Curtis Puryear, Chelsea Schein, Kurt Gray

**Affiliations:** ^a^Department of Psychology, University of Koblenz-Landau, 76829 Landau, Germany;; ^b^Department of Psychology and Neuroscience, University of North Carolina at Chapel Hill, Chapel Hill, NC 27599;; ^c^Department of Legal Studies and Business Ethics, The Wharton School of Business, University of Pennsylvania, Philadelphia, PA 19104

**Keywords:** morality, politics, poltical tolerance, moral psychology, narrative

## Abstract

Both liberals and conservatives believe that using facts in political discussions helps to foster mutual respect, but 15 studies—across multiple methodologies and issues—show that these beliefs are mistaken. Political opponents respect moral beliefs more when they are supported by personal experiences, not facts. The respect-inducing power of personal experiences is revealed by survey studies across various political topics, a field study of conversations about guns, an analysis of YouTube comments from abortion opinion videos, and an archival analysis of 137 interview transcripts from Fox News and CNN. The personal experiences most likely to encourage respect from opponents are issue-relevant and involve harm. Mediation analyses reveal that these harm-related personal experiences increase respect by increasing perceptions of rationality: everyone can appreciate that avoiding harm is rational, even in people who hold different beliefs about guns, taxes, immigration, and the environment. Studies show that people believe in the truth of both facts and personal experiences in nonmoral disagreement; however, in moral disagreements, subjective experiences seem truer (i.e., are doubted less) than objective facts. These results provide a concrete demonstration of how to bridge moral divides while also revealing how our intuitions can lead us astray. Stretching back to the Enlightenment, philosophers and scientists have privileged objective facts over experiences in the pursuit of truth. However, furnishing perceptions of truth within moral disagreements is better accomplished by sharing subjective experiences, not by providing facts.

## Significance

All Americans are affected by rising political polarization, whether because of a gridlocked Congress or antagonistic holiday dinners. People believe that facts are essential for earning the respect of political adversaries, but our research shows that this belief is wrong. We find that sharing personal experiences about a political issue—especially experiences involving harm—help to foster respect via increased perceptions of rationality. This research provides a straightforward pathway for increasing moral understanding and decreasing political intolerance. These findings also raise questions about how science and society should understand the nature of truth in the era of “fake news.” In moral and political disagreements, everyday people treat subjective experiences as truer than objective facts.

Political animosity in the United States is extremely high (1), with liberals and conservatives frequently disrespecting and demonizing each other (2, 3). In politics today, both elected officials (4) and everyday people (5) are reluctant to engage with political opponents. This political intolerance undermines open discussion and debate—a cornerstone of civic institutions (6)—and may blind us to the common humanity of political opponents (7). Is there a way to increase respect across the political divide?

Past research reveals a number of potential strategies for dampening intergroup intolerance, such as emphasizing superordinate goals (8) or providing intergroup contact (9). However, these strategies are difficult to implement because they require repeated positive interactions among opponents; single one-off interactions with outgroup members tend to lead to more intolerance, not less (10). Creating multiple opportunities for positive cross-politics interaction is especially challenging in modern America because conservatives and liberals live in different communities (11) and are spending less time together (12). Here, we investigate how even single one-off interactions might foster respect between political opponents.

We suggest that increasing perceptions of opponents’ rationality might help bridge the political divide, which we define as fostering respect for people with opposing political viewpoints. Decades of research highlights the link between outgroup antagonism and seeing outgroup members as mindless or irrational (13). Those who disagree with our strongly held beliefs often seem brainwashed or ignorant (14), which helps explain why discussions with political opponents often seem unproductive (15). Increasing the perceived rationality of political opponents could, therefore, increase political tolerance.

How might perceptions of rationality be increased? We examine two different strategies for interactions between political opponents—supporting one’s moral beliefs with facts (objective statistics and evidence obtained from reports and articles) versus personal experiences (subjective anecdotes about lived events).

## People (Mistakenly) Believe that Facts Foster Respect

Ever since the Enlightenment (16), definitions of rationality have emphasized the importance of truth (17) and logic (18) in forming conclusions. It seems definitional that rational people should privilege facts (19) and see objective data and statistics as “truer” than subjective personal experiences (20). Accordingly, grounding your political position in facts would seem to be essential to establishing your rationality, which in turn should foster respect from political opponents.

**Fig. 1.**
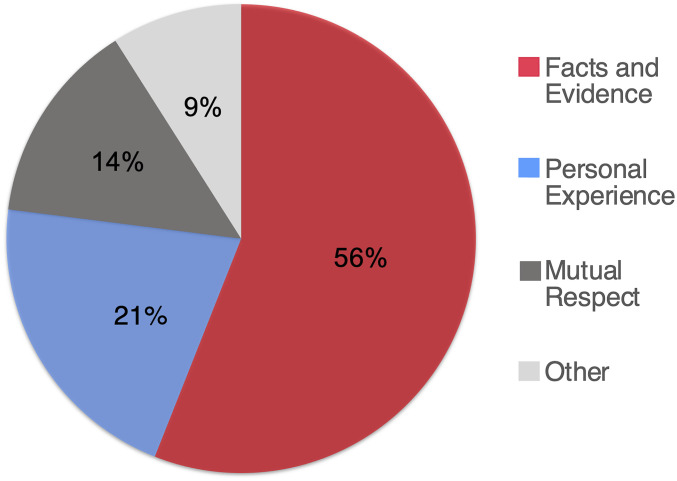
Categorized free responses of participants answering the prompt “I would respect their opposing opinion if it was based upon….” (Study 1).

Reflecting the longstanding importance of facts in philosophical and scientific discourse, the American public also believes that facts are the pathway to increasing respect in moral/political discourse. Using a free-response format, participants (Study 1*, *n* = 251) were asked to “imagine someone disagrees with you on moral issues” (e.g., same-sex marriage or abortion) and “what would make you respect their opinion?” Responses were categorized into themes with a majority of respondents (55.78%) stating that basing one’s stance on facts and statistics would increase respect, followed by basing one’s stance on personal experiences (21.12%), followed by an understanding of mutual respect (14.34%). Significantly more participants believed facts and evidence would increase respect as compared to personal experience (χ^2^ = 63.26, *P* < 0.001; see Fig. 1).

We next asked a representative sample of participants (Study 2, *n* = 859) to imagine interacting with two political opponents, one who based their beliefs in facts and statistics and one who based their beliefs in personal experiences. Participants rated the opponent who based their stance on facts as more rational than the opponent who based their stance on personal experiences (facts: mean [M] = 5.41, SD = 1.32; personal experience: M = 4.58, SD = 1.30; *t*(858) = 15.55, *P* < 0.001), respected them more (facts: M = 5.58, SD = 1.20; personal experience: M = 4.99, SD = 1.25; *t*(858) = 13.41, *P* < 0.001), and wanted to interact with them more (facts: M = 5.69, SD = 1.15; personal experience: M = 5.14, SD = 1.26; *t*(858) = 13.50, *P* < 0.001).

When imagining abstract political discourse, everyday Americans believe that supporting one’s beliefs with facts leads to respect; however, the effectiveness of facts is unclear in concrete cases, such as when arguing with a stranger about gun rights. The problem is that facts—at least today—are themselves subject to doubt, especially when they conflict with our political beliefs (21). In the past decades, America has seen a decentralization of news and information (22) that has allowed people to gather their “own facts” (i.e., alternative facts; ref. 23). Most recently, claims of “fake news” allow people to distrust any information that fails to align with their political beliefs (24, 25) and to trust fake news that aligns with their beliefs (26).

## The Power of Personal Experience

The current distrust of facts suggests that the route to perceived rationality and respect may paradoxically lie in personal experience. Statistics can be doubted and countered with other statistics, but first-hand experiences have an aura of unimpeachability. To paraphrase the philosopher Kierkegaard, truth is not something to be viewed objectively but instead to be subjectively experienced (27). Consistent with this idea, evolutionary accounts argue that our minds have evolved to process personal narratives (28) and be persuaded by stories (29). This may be especially true in morality and politics. People may be open to objective facts in many domains (e.g., buying cars, choosing investments), but perhaps not when it comes to matters of politics or morality.

**Fig. 2.**
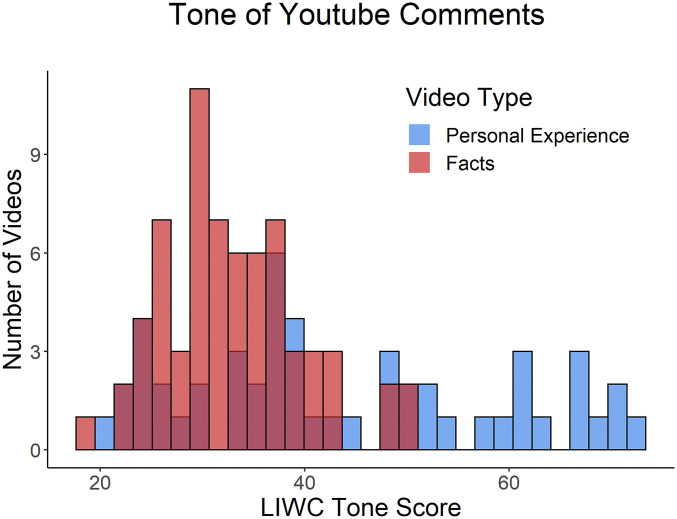
Histogram of emotional tone scores (i.e., the average linguistic inquiry word count [LIWC] tone score for all comments on a video) for the 51 experience and 67 facts/evidence abortion opinion videos (Study 3).

As an initial test of how much facts versus personal experiences predict respectful discourse, we used a social media platform known for disrespect—YouTube—where we collected comments surrounding a concrete controversial issue (abortion; Study 3). Using predefined terms for video collection, we examined all 300,978 comments for 194 YouTube videos expressing opinions about abortion—videos that either emphasized facts (e.g., informational videos from websites like *Buzzfeed* and *NowThisNews*) or personal experiences (e.g., people telling stories about their abortions on a webcam or on the news). We then conducted a text analysis of the comments associated with these videos. Analyses revealed that, compared with videos providing facts, videos that shared personal experiences had comments with more positive emotion words (Beta [*B*] *=* −3.20, SE = 0.42, *P* < 0.001) and an overall more positive emotional tone (*B* = −10.55, SE = 1.90, *P* < 0.001) (Fig. 2). Comments on personal experience videos were also significantly more likely to include affiliative words (i.e., social and ally based; *B* = −0.89, SE = 0.15, *P* < 0.001). These results suggest that beliefs about the power of facts to foster respect may be mistaken.

**Fig. 3.**
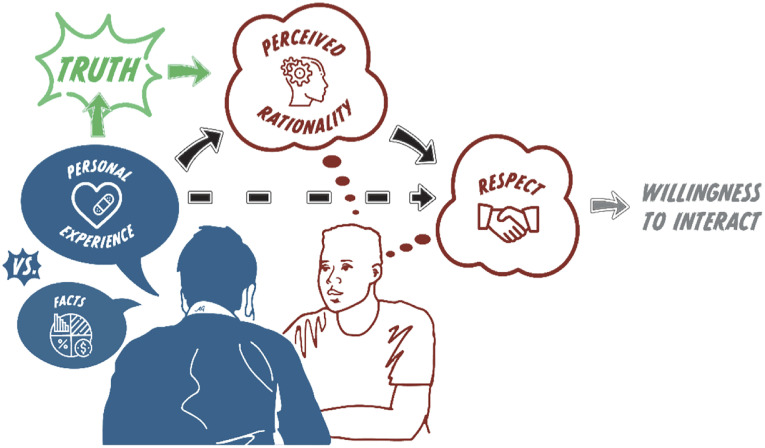
A theoretical model. The core model is joined by black arrows: basing one’s moral stance in personal experience (versus facts) fosters more respect in political opponents (Studies 4 through 15). This link from personal experience to respect is mediated by increased perceptions of rationality. The gray arrow traces an additional prediction: that increased respect toward political opponents predicts an increased willingness to interact with them. The green arrow traces a prediction about the perceived nature of truth: personal experiences seem truer (i.e., are doubted less) within moral disagreements, which predicts increased perceived rationality (Studies 13 and 15). Figure credit: Avital Glibicky.

Here, we systematically compare whether people are more respectful of the conflicting moral beliefs of political opponents when they are based in either facts or personal experiences. Fig. 3 details the predicted core psychological model: basing one’s moral beliefs in personal experience increases perceived rationality, which in turn increases respect. We argue that fostering respect is not only important in itself but also predicts an increased willingness to interact with opponents. We also suggest that, within moral/political disagreements, perceptions of rationality are increased more by personal experiences than facts because personal experiences seem “truer” (i.e., are doubted less). In total, we present 15 studies which include various methods, samples, and political/moral issues. All studies are presented in full in the *SI Appendix*.

Personal experiences are as rich and varied as there are people on Earth and days in a year; here, we examine the tolerance-inducing potential of personal experiences involving harm. In many real-world cases, people cite their own suffering or that of a close other as instrumental in motivating political action. For example, experiencing, expecting, or witnessing harm prompted school shooting survivors to support gun control (30), prompted young Black people to support BlackLivesMatter (31), and prompted coal miners to support reduced environmental restrictions (32). Beyond these examples, harm-based narratives are extremely well represented in political discourse and everyday life (33, 34).

Harm-based personal experiences may be especially powerful in fostering respect because, as Darwin long ago recognized, it is rational for any organism to avoid harm (35). Harm is also a central consideration in morality and politics (36). The emerging Theory of Dyadic Morality (37) suggests that the vast majority of moral judgments—for both liberals and conservatives (38)—revolve around intuitive and culturally situated perceptions of harm. Tying political viewpoints to harm (or its possibility) therefore provides a common currency for political discussions and lays the groundwork for mutual moral understanding. Past work shows how personal experiences of harm can induce sympathy (39), especially when harm befalls those who are identifiable (40) and similar to close others (41). However, sympathizing with someone is not the same as respecting them. In fact, when people sympathize with someone, they tend to view them as less rational (42, 43).

Studies in both psychology and political science support the idea that personal narratives can help persuade. Narratives can “transport” people away from the here-and-now (44), and stories that facilitate attentional absorption can help to change political beliefs (45). One large-scale study found that narratives, when paired with nonjudgmental listening, can shift views on contentious issues (46). The rich literature on political persuasion (47, 48) further highlights the ability for narratives to persuade (49)—often because narratives typically present information “peripherally,” minimizing the likelihood for counterarguments from “central” processing (50).

In contrast to past work, we focus primarily on building respect and not on persuasion. Although these two outcomes are certainly related—persuasion typically requires a foundation of interpersonal respect (51)—these constructs are not identical; it is possible to respect another person without agreeing with their moral beliefs. That being said, persuasion is often a goal of political discussions (52), but some scholars argue that political discourse is most productive when we first focus on creating mutual respect (53, 54). Of course, even with the goal of building respect, trying to persuade others is part of human nature (55), but moral convictions are extremely difficult to change (56). Here, we leave aside the question of persuasion to focus on how best to foster respect in moral disagreements with political opponents.

## Testing the Core Model: From Personal Experiences to Perceived Rationality to Respect

In political disagreements, does basing one’s moral beliefs on personal experiences foster respect more than basing one’s moral beliefs on facts, and does it do so via perceptions of rationality? In the first test of the core model sketched out in Fig. 3 (Study 4, *n =* 177), participants reported their stance on tax, coal, and gun policy and then read about individuals who disagreed with them on these subjects—either due to personal experiences (e.g., lost his job due to new coal regulations) or factual knowledge (e.g., facts he learned while reading about the topic). Participants then rated how rational the opponent seemed (e.g., “is logical for holding their stance”), how willing they were to respect the opponent (e.g., “be considerate of this person’s point of view”), and how willing they were to interact with them (e.g., “exchange ideas with this person”).†

Results indicated that stances based on personal experiences increased perceived rationality (M = 4.78, SD = 1.26) more than stances based on facts (M = 4.13, SD *=* 1.29), *t*(175) = 0.02, *P* = 0.001, *d =* 0.51. Personal experiences also fostered more respect (M = 4.90, SD = 1.17) than facts (M = 4.46, SD = 1.15; *t*(175) = 0.23, *P* = 0.01, *d* = 0.38). As predicted, perceived rationality mediated the relationship between personal experiences (versus facts) and respect (indirect effect = 0.48, SE = 0.14, 95% CI [0.20, 0.78]).

We note that the indirect effect from personal experience to willingness to interact via rationality/respect is significant in every study (*SI Appendix*). However, in the main text, we only report when personal experience has a significant total effect on willingness to interact. Meta-analyses of our data (presented near the end of the paper) reveal a significant total effect of personal experiences (compared to facts) increasing both respect toward political opponents and people’s willingness to interact with them.

In this and all other studies, we tested for interaction effects between condition and participant stances on political issues (e.g., pro–gun rights versus pro–gun control) for ratings of rationality and respect. No interaction effects were significant,^‡^ and participant political ideology did not moderate indirect effects (*SI Appendix*), revealing the power of personal experience for both liberals and conservatives.

Study 5 (*n =* 153) tested our model in face-to-face conversations about guns. Each participant was recruited from a public location in Chapel Hill or Durham, North Carolina, and spoke with someone they thought was another passerby but who was actually a member of the research team who memorized a series of conversational prompts. This research assistant took the opposing stance on gun policy to the participant and offered either personal experiences or shared factual knowledge to support that stance.§ We recorded the verbal responses of the participant, which were then parsed and rated by blind-to-condition coders.

Analyses of the conversations revealed that opponents who based their stance on personal experiences were treated as more rational (*t*(151) = −3.71, *P* < 0.001), were respected more [*t*(151) = −3.26, *P* = 0.001], and participants were more willing to interact with them [*t*(151) = −3.57, *P* < 0.001], as compared to opponents who based their stance on facts. As predicted, personal experience predicted respect via perceived rationality (indirect effect = 0.56, SE = 0.16, 95% CI [0.26, 0.90]) and subsequently predicted willingness to interact (indirect effect = 0.41, SE = 0.13, 95% CI [0.18, 0.70]) through perceived rationality. In both vignettes and real face-to-face conversations, basing one’s personal experiences (versus facts) in political discussions increased perceived rationality, which in turn fostered respect.

Study 6 (*n =* 194) sought to replicate the previous studies while ruling out the possible alternative explanation that personal experiences are more specific or concrete. We contrasted real and concrete facts, taken from https://www.justfacts.com/guncontrol.asp (e.g., “someone reads in an annual report that 73% of murders in the United States are committed with firearms), with personal experiences (e.g., “someone’s young daughter is hit by a stray bullet”) in a vignette experiment similar to Study 4. A pilot study found that these facts were rated as higher in specificity and concreteness than the personal experiences. Despite this difference, personal experiences again fostered respect more than facts, as mediated through perceived rationality (indirect effect = 0.58, SE = 0.13, 95% CI [0.34, 0.85]). These results suggest that the power of personal experiences (versus facts) to increase perceived rationality and foster respect is not driven by its greater specificity or concreteness.

## What Kind of Experiences Best Foster Respect? Relevant, Harm-Based, and Personal

The previous studies reveal that basing an opinion on personal experiences fosters respect better than basing one’s stance on facts. However, open questions remain about exactly which personal experiences best foster respect. We have suggested that “relevant,” “harm-based,” and “personal” experiences are maximally respect-inducing, but this remains to be tested. In three studies about guns, we tested the importance of each of these aspects of experience: relevant (Study 7), harm-based (Study 8), and personal (Study 9).

Study 7 (*n =* 273) tested whether personal experiences need to be relevant to the topic of disagreement to foster respect. Our data reveal that perceptions of rationality are crucial for respect, and so relevance would presumably be important—it seems irrational to ground your moral/political beliefs in irrelevant personal experiences (e.g., I am pro–gun rights because I was harmed by corporate tax policy). On the other hand, it may be that the mere existence of any personal suffering could increase respect. In this vignette study, participants rated opponents who based their stance on relevant facts about guns, relevant personal experiences with guns,^¶^ or nonrelevant personal experiences (i.e., had their business go into bankruptcy). Results revealed that relevant personal experiences were significantly better at increasing perceived rationality (M = 4.86, SD = 1.60) and respect (M = 5.19, SD = 1.26) compared to both facts (rationality: M = 3.74, SD = 1.40, *P* < 0.001; respect: M = 4.40, SD = 1.34, *P* < 0.001) and nonrelevant personal experiences (rationality: M = 3.60, SD = 1.42, *P* < 0.001; respect: M = 4.29, SD = 1.41, *P* < 0.001). There was no significant difference between facts and nonrelevant personal experiences. These results suggest that personal experiences need to be relevant to the issue at hand to foster respect between opponents.

Study 8 (*n* = 255) tested whether personal experiences should involve harm to best foster respect from political opponents. This vignette study compared ratings of opponents who based their stances on harm-based personal experiences (e.g., used a gun to protect my family from an intruder), nonharm personal experiences (e.g., took a firearm safety course), or facts. Results revealed that harm-based personal experiences were more effective at increasing perceived rationality (M = 5.00, SD = 1.42) and respect (M = 5.34, SD = 1.21) relative to nonharm personal experiences (rationality: M = 4.09, SD = 1.57, *P *< 0.001; respect: M = 4.55, SD = 1.41, *P* < 0.001). However, nonharm (but still relevant) experiences were significantly more effective at increasing perceived rationality and fostering respect compared to facts (rationality: M = 3.44, SD = 1.62, *P* = 0.007; respect: M = 3.95, SD = 1.65, *P* = 0.008). These results highlight the general weakness of facts in political disagreements, as any relevant personal experience is more effective than facts for bridging divides.

Study 9 (*n* = 408) tested whether personal experiences need to be “personal” to foster respect from political opponents. This vignette study compared ratings of opponents who held their stance due to a harm-based experience at three levels of personalness: high (they themselves suffered), moderate (they have a friend or relative who suffered), or low (they read about someone who suffered). The results revealed the predicted “personalness gradient,” with more “personal” experience fostering more respect (high personal experience: M = 5.08, SD = 1.29; moderate: M = 4.66, SD = 1.36; low: M = 4.21, SD = 1.29); *F*(4,403) = 4.79, *P* = 0.001; see *SI Appendix* for more details).

## Generalizing the Effect to Real-World Settings

Together, the previous three studies reveal that relevant, harm-based, and personal experiences are most effective at bridging political and moral divides. However, open questions remain because many of our past studies use vignettes constructed by the authors and rely on convenience samples (i.e., Amazon Mechanical Turk, or MTurk). Study 10 (*n* = 1,565) sought to replicate and generalize our past studies using a representative sample with real gun policy statistics (from *Every Town for Gun Safety* and *National Rifle Association*), such as “murder rates were 19.3% higher when the Federal assault weapon ban was in effect.” We also collected real gun-related personal experiences (solicited from another set of participants) and, based on additional pilot testing, divided these personal experiences into three sets—low harm, medium harm, and severe harm^#^—to provide another test of the importance of harm.

**Fig. 4.**
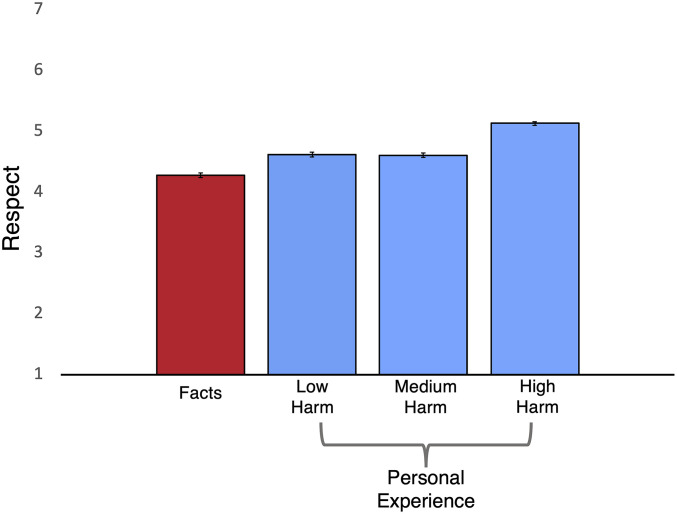
Average ratings of respect across conditions (Study 10). Post hoc analyses revealed that fact-based beliefs were significantly less respected than all three types of personal experience (low harm; *P* = 0.03, medium harm; *P* = 0.004, and high harm; *P <* 0.001). Error bars represent SEs.

Participants read about a political opponent who based their moral beliefs either on a real personal experience or a real statistic. Compared to opponents who provided facts to justify their stance, providing personal experiences (across all levels of harm) increased perceived rationality (personal experience: M = 4.13, SD = 1.61; facts: M = 3.79, SD *=* 1.58; *t*(1558) = −4.21, *P* < 0.001) and fostered more respect (personal experience: M = 4.71, SD *=* 1.43; facts: M = 4.28, SD *=* 1.45; *t*(1561) = −5.93, *P* < 0.001). Consistent with predictions about the importance of harm, personal experiences high in harm were most likely to increase respect and perceived rationality (see Fig. 4). High-harm personal experiences also increased participants’ willingness to interact with opponents relative to facts (personal experience: M = 5.52, SD = 1.35; facts: M = 5.16, SD = 1.49; *t*(1060) = −3.38, *P* = 0.001). Mediation analyses showed that personal experience fostered respect through perceptions of rationality (indirect effect = 0.21, SE = 0.05, 95% CI = [0.11, 0.31]).

The past studies reveal that personal experiences bridge divides better than facts, but many of these were tightly controlled manipulations. Although this control makes it easier to draw causal inferences, it poses challenges for generalizability. Of course, the YouTube comments study (Study 3) reveals the tolerance-inducing power of personal experiences in social media, but to further test the generalizability of our effects, we examined two real-world stimuli/situations: *New York Times* op-eds (Study 11) and interviews between opponents on CNN and Fox News (Study 12).

Study 11 (*n =* 425) examined *New York Times* articles that varied naturalistically in how much they emphasized facts and personal experiences. Four op-eds were selected—two pro–gun control and two pro–gun rights—and one of each of these emphasized either facts or personal experience (as confirmed by pilot testing). Participants read one op-ed of an opponent (either facts or personal experience) and then rated the op-ed author on rationality, respect, and willingness to interact. Analyses revealed that authors who shared personal experiences rather than provided facts were seen as more rational (personal experience: M = 4.95, SD = 1.56; facts: M = 4.13, SD = 1.84; *t*(423) = −4.99, *P* < 0.001, *d* = 0.48), were respected more (personal experience: M = 5.43, SD = 1.24; facts: M = 4.61, SD = 1.73; *t*(423) = −5.60, *P* < 0.001, *d =* 0.54), and participants were more willing to interact with them (personal experience: M = 5.70, SD *=* 1.27; facts: M = 5.15, SD *=* 1.68; *t*(423) = −3.78, *P* < 0.001, *d =* 0.37). Mediation models revealed that personal experience led to greater respect via perceived rationality (indirect effect = 0.54, SE = 0.12, 95% CI [0.31, 0.78]) and respect predicted willingness to interact (indirect effect = 0.31, SE = 0.08, 95% CI [0.17, 0.47]).

Study 12 examined 137 transcripts of interviews on CNN and Fox News between political opponents—that is, when CNN hosts interviewed conservative guests and when Fox hosts interviewed liberal guests. The goal of this study was to provide greater generalizability by examining the effectiveness of facts and experiences used in real-world political discussions. Transcripts were collected from 2002 to 2004, 2008 to 2010, and 2015 to 2017 to provide generalizability across time. Transcripts were then coded by research assistants on a variety of measures (*SI Appendix*). The key independent variable of interest was how much the guest (i.e., interviewee) shared personal experiences versus facts, as measured as a difference score. Note that mentioning personal experiences and facts were inversely related (*r*(135) = −0.49, *P* < 0.001), suggesting that these two justifications for moral beliefs may often be in competition. Guests who shared experiences rather than facts were treated as more rational by hosts (*r*(135) = 0.20, *P* = 0.02). The total effect of personal experience (versus facts) on respect was not significant, although mediation analyses revealed the predicted pattern from experiences (versus facts) to respect via perceived rationality (indirect effect = 0.06, SE = 0.03, 95% CI = [0.01, 0.12]).

## In Moral Disagreement, Experiences Seem Truer than Facts

Across many studies, basing one’s stance on personal experiences (versus facts) seems to make people appear more rational to opponents. We suggest that this effect is because personal experiences are unimpugnable; first-hand suffering may be relatively immune to doubt. Although the very nature of truth seems objective, research reveals how its perception is influenced by extraneous factors (57). The next study examined the “truth perception” of personal experiences versus facts. Facts may technically be “objective,” but in the modern world, someone can always counter one fact with another, making facts easily doubted. In contrast, personal experiences may be harder to doubt, especially in the domain of morality where subjective beliefs seem objective (58). If personal experiences are treated as “truer” and doubted less, it would help explain why personal experiences increase perceived rationality—subjectively speaking, personal experiences of harm are actually more “objective.”

In Study 13 (*n* = 508), we explored the importance of perceived truth (and its inverse, doubt) in our core model. We predicted that people may be especially likely to doubt the truth of facts provided by opponents in moral disagreements. We presented participants with a target who either disagreed or agreed with them and who based their beliefs on either personal experiences or facts that supported their beliefs. This disagreement or agreement was either within a moral (e.g., gun policy) or nonmoral context (e.g., blender preferences). This study, therefore, had eight conditions: 2 (agree versus disagree) × 2 (moral versus nonmoral) × 2 (personal experience versus fact). After reading about the target, participants rated how much they doubted the truth of the target’s statement before rating perceived rationality, respect, and their willingness to interact.

**Fig. 5.**
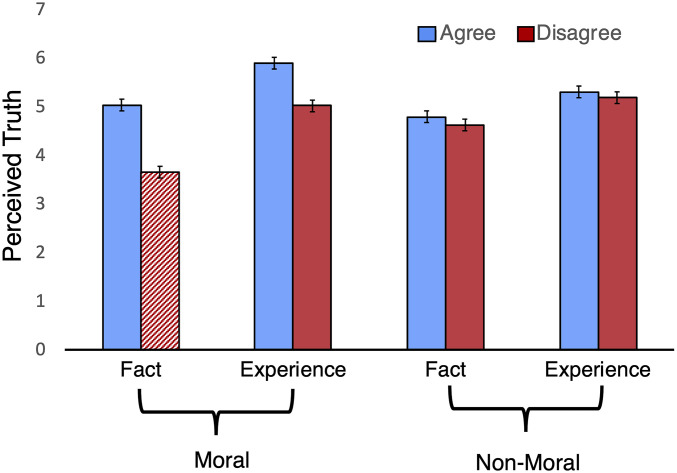
The mean ratings of perceived truth (i.e., lower scores = higher doubt) across conditions (Study 13). Error bars represent the SEs. The red hatched bar shows much lower levels of perceived truth in the fact/disagree/moral condition. Error bars represent SEs.

Planned comparisons using Bonferroni corrections revealed that doubt (questioning the truth of a statement) was highest in one condition: when opponents based their stance on facts in matters of morality/politics (Fig. 5). Consistent with our model, specific doubts about “moral facts” (versus moral personal experiences) mediated the key effect found throughout these studies: personal experiences (versus facts) leading to increased perceptions of rationality in political opponents (indirect effect = 0.81, SE = 0.10, 95% CI [0.62, 1.00])—which, in turn, predicted increased respect. In morality and politics, facts themselves are subject to doubt and thus fail to furnish perceptions of rationality in opponents. Conversely, personal experiences seem true even among opponents who disagree with the views supported by those experiences.

## Whose Experiences Foster Respect? Examining Potential Boundary Conditions

Despite the consistent findings of our past studies, lingering questions remain about generalizability and alternative explanations. In our last two studies, we sought to generalize our effects to two different targets—a woman of color (Study 14) and a scientist discussing their research (Study 15). Study 14 also included a number of methodological tweaks to further rule out alternative explanations, and Study 15 examined a new issue—immigration—to provide additional generalizability.

The first goal of Study 14 (*n* = 1,897) was to examine the personal experiences of women and people of color (59), whose accounts of harm are often dismissed (60), especially when they challenge the political status quo (61, 62). Second, we wanted to compare the effect of facts versus personal experience to a strict control condition in which opponents simply asserted their political stance without using either facts or personal experiences to support it.^‖^ This allowed us to test whether using facts in political disagreements was at least better than providing no justification. Third, we wanted to better equate the language across conditions. In the previous studies, political opponents who based their stance on facts explicitly mentioned the source of those facts (e.g., read in a governmental report), which could have seemed awkward and made facts seem less credible. We revised the vignettes in this study to not include such language. Fourth, we wanted to ensure that the respect-inducing power of personal experience was not because people saw these experiences are more specific, evocative, or salient, which we accomplished by providing especially potent real facts.

In this study, participants learned about a Black woman who disagreed with their stance on gun policy (i.e., whether there should be stronger or weaker gun regulations) and who supported her beliefs by providing either personal experiences, facts, or no rationale at all (this acted as our control condition). Pretesting revealed that the facts were rated as either higher or not significantly different on specificity, salience, and evocativeness compared with the personal experience condition (*SI Appendix*), which ensured that these dimensions could not easily explain the ability of personal experiences to increase respect.

Results revealed that the target who based their stance on personal experiences was seen as significantly more rational (M = 5.05, SD *=* 1.42) and was more respected (M = 5.40, SD = 1.15) compared to both a target who provided no rationale (rationality: M = 3.84, SD *=* 1.40; respect: M = 4.70, SD = 1.33) and a target who provided facts (rationality: M = 4.66, SD *=* 1.48; respect: M = 5.05, SD = 1.25); all comparisons were significant at *P* < 0.001. Mediation analyses revealed that personal experiences (versus facts) fostered respect through perceived rationality (indirect effect = −0.23, SE = 0.05, 95% CI [−0.23, −0.02]). The total effect of the model was significant as well (*b* = −0.35, SE = 0.07, 95% CI [−0.49, −0.21]).

These results replicate key findings from our past studies: basing one’s stance in personal, relevant, harm-based experiences fosters more respect than basing one’s stance in facts, which fosters more respect than providing no rationale at all. This study also provides initial evidence that the respect-inducing power of personal experiences may extend to the experiences of women of color, although much more research is necessary to fully explore this question.

Our past studies have reliably revealed that experiences are better than facts at bridging divides. However, these studies all examined laypeople as targets, and it may be easy to doubt facts when they are provided by the average American. It may be more difficult to doubt facts provided by scientists, not only because of their professional standing but because scientists often have first-hand experience in collecting these facts.

In this final study, we used a representative sample to test how people perceive scientists as political opponents (Study 15, *n* = 1,412). This study examined a different issue than previous studies—immigration policy—to help us further generalize our results. There were three conditions; participants read about a layperson who based their opposing stance on personal experience, a layperson who based their opposing stance on factual knowledge, or a scientist who based their opposing stance on their own scientific research (a grant-funded project that examined the impacts of immigration policy). This study examined the full model (see Fig. 3) of doubt, perceived rationality, respect, and willingness to interact using measures from the previous studies.

The results revealed that, across all three conditions, participants saw the personal experiences of the layperson as the “truest,” followed by scientific research, and then facts provided by a layperson. That people see one person’s anecdotal experience as truer than the conclusions of scientific research is striking. Interestingly, despite these differences in perceived truth, scientists themselves were deemed quite rational, and these perceptions translated into respect—perhaps welcome news for those who do science (Table 1). However, scientists were still not respected as much as someone with relevant, harm-based experience.

**Table 1.** ANOVA analyses of dependent variables between the three conditions of the scientist study (Study 15)

Condition	Doubt	Rationality	Respect	Interact
	Mean (SD)	Mean (SD)	Mean (SD)	Mean (SD)
Experience	4.77 (1.44)^a^	4.02 (1.71)^a^	4.64 (1.47)^a^	5.49 (1.31)^a^
Scientist	4.47 (1.36)^b^	4.20 (1.54)^a^	4.46 (1.48)^b^	5.56 (1.34)^a^
Fact	3.75 (1.45)^c^	3.61 (1.62)^b^	4.04 (1.47)^c^	5.20 (1.34)^b^

Means without a superscript letter in common are significantly different from each other at *P* < 0.05. Higher scores on the doubt measure indicated less doubt (i.e., greater perceived truth).

Mediational analysis revealed that personal experience (as compared to scientific research) led to increased rationality and subsequent respect through the pathway of perceiving personal experiences as truer (i.e., less doubted) than scientific research (indirect effect = −0.11, SE = 0.04, 95% CI [−0.18, −0.04]). We also examined two moderators of this mediation pathway: participant ideology and belief in science. Personal experience fostered more respect than scientific research regardless of ideology, but participants with strongly held beliefs about the value of science respected scientists just as much as targets with personal experience (*SI Appendix*). While scientific expertise does help build respect compared to just providing facts, harm-based personal experience remains the most effective route to respect across all Americans.

**Fig. 6.**
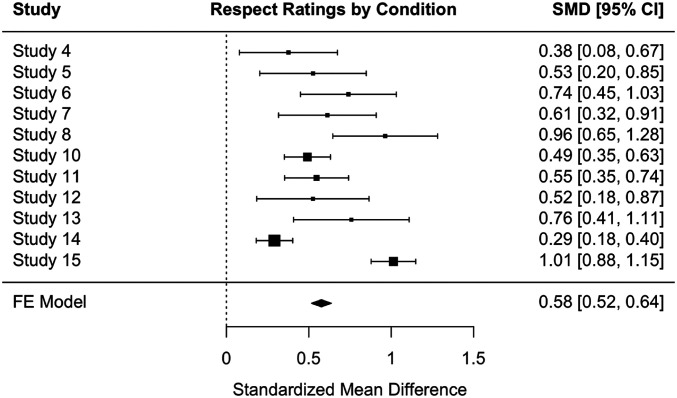
A forest plot of meta-analysis of ratings of respect toward a political opponent by condition (personal experience versus facts) across studies.

**Fig. 7.**
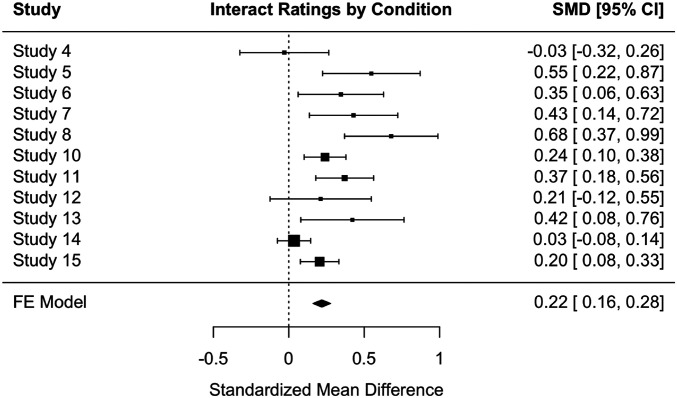
A forest plot of meta-analysis of ratings of willingness to interact with a political opponent by condition (personal experience versus facts) across studies.

As a final assessment of the effects observed across all 15 studies, we conducted two meta-analyses: one on the effectiveness of personal experience (versus facts) in fostering respect and one on personal experience (versus facts) in increasing the willingness to interact with political opponents. Included were all studies that used both personal experiences and facts (all but Studies 1 through 3 and 9). These meta-analyses found a robust effect for personal experience on both respect (Hedge’s *g* = 1.86) and willingness to interact (Hedge’s *g* = 1.07). The fixed effects models further suggested significant effects on both (respect: B = 0.58, SE = 0.03, 95% CI [0.52, 0.64]; willingness to interact: B = 0.22, SE = 0.03, 95% CI [0.16, 0.28]) (Figs. 6 and 7).

## General Discussion

Across 15 studies, our results shed light on the nature of political divides and how to better bridge them. People explicitly believe that facts and statistics are important when trying to build mutual respect, but the reality is that personal experiences are better. Because personal experiences are seen as truer than facts, they furnish the appearance of rationality in opponents, which in turn increases respect. Not all personal experiences are created equal, however. The most effective personal experiences are those that are both relevant to the issue (Study 7) and involve harm—suffering or potential suffering (Study 8)—likely because much of morality is grounded in perceptions of harm (37). It is also important to note that personal experiences only have an advantage in moral disagreement—for agreement (whether moral or nonmoral) and for nonmoral disagreement, facts foster respect just as well as experiences (Study 13).

Of course, there are limitations in this work. We acknowledge that these studies often used self-report data from online samples; however, this research also included archival analyses in social media and traditional media, behavioral research with community members, and representative samples. It is an open question of whether these results apply cross-culturally, but we suggest that it may be useful to improve respect among the citizens of even one politically fractured country.

Future work should examine moderators of these results, continuing to explore whether the perceived truth of experiences hinges upon the identity of the person sharing them. The experiences of people belonging to racial and sexual minorities have long been doubted to thwart social change. Although the targets in our vignettes varied in race and gender (see Study 14), we did not specifically test whether personal experiences with sexism or racism foster respect more than facts. It is noteworthy, however, that powerful movements for social change (e.g., #MeToo, #BlackInTheIvory) rely heavily on sharing personal experiences. Additionally, we did not test whether learning about personal experiences (versus facts) is more or less effective at fostering respect when people lack strong preexisting opinions. People who have spent less time thinking about an issue may be less skeptical of facts, but then again, people who care little about an issue may still find personal narratives compelling.

In this paper, we have contrasted facts and personal experience as opposing strategies, consistent with longstanding philosophical (63) and scientific discussions (64) about epistemology. The tension between facts and personal experience is also revealed by the inverse correlation between them in our data (e.g., Study 12). However, the most productive conversations may involve some combination of both personal experiences and facts. We speculate that personal experiences might be deployed early in conversations to first build a foundation of mutual respect, and then facts could be introduced as the conversation moves to policy specifics.

These studies examined everyday Americans, and it is unclear whether the benefits of sharing personal experiences extends to interactions between elected officials, or improves the quality of policies they implement. Facts certainly matter for creating policy solutions, but creating effective bipartisan solutions requires that political opponents work together, and it is easier to work with someone you respect—as recent initiatives to bridge divides among politicians illustrate (65). Providing relevant personal experiences may also be an effective strategy for building respect among policymakers who likely need to build a common understanding before appreciating the validity of opposing facts.

These results provide a way to bridge moral divides. Many researchers have explored polarization between liberals and conservatives (66, 67) and its downstream consequences (68), but fewer scholars have revealed scalable mechanisms to overcome this polarization. These results suggest that sharing personal experiences may be one route to help build moral understanding between political opponents. Using anecdotes to ground political discussions may feel flimsy in the age of modern science, but some argue (69) that the constant deluge of data and the rise of social media have moved us to the era of “post-truth.” Of course, appreciating facts and statistics is essential for effective governance, and because of this, many people—especially scientists—have been taught to value facts above all else in the pursuit of truth. However, when people deeply believe in opposing moral values, facts seem untrue and therefore fail to create political tolerance. On the other hand, personal experiences furnish perceptions of both truth and rationality in political opponents, leading to mutual respect.

## Supplementary Material

Supplementary File

## Data Availability

Raw data and materials are available at Open Science Framework (https://osf.io/kbvmn/).

## Footnotes

*All studies (except Study 2) were preregistered. Links to preregistrations can be found in *SI Appendix*. Furthermore, all studies were approved by the University of North Carolina at Chapel Hill Institutional Review Board (IRB). Study 5 was approved under IRB number 18-1182; all other studies were approved under IRB number 18-0177. All participants provided informed consent.

^†^In all subsequent studies, measures of rationality, respect, and interaction were identical. In studies with coders rating the dependent variables, codes were based on these measures.

^‡^Interaction effects were tested in all other studies except Study 12, as it was not possible based on the study design. See *SI Appendix*. Interaction analyses were not expected and not preregistered.

^§^Given that the recruitment area traditionally votes for Democrats, the confederate was “pro–gun rights” 97% of the time.

^¶^The relevant and harmful personal experiences conditions used in Studies 7 and 8 were identical to the personal experience conditions used in Study 4.

^#^Quotes were taken verbatim from participants.

^||^We also ran a study that was partly motivated by this same goal, focusing on a new context (the Black Lives Matter versus Blue Lives Matter movements), which replicated our effects. However, due to some interpretation concerns (caused by confounds across vignettes), we only report these findings in *SI Appendix*.
