# Supplementary material for: Personal experiences bridge moral and political divides better than facts
Source: Proc Natl Acad Sci U S A. 2021 Jan 25;118(6):e2008389118. doi: 10.1073/pnas.2008389118 (PMC8017692; doi:10.1073/pnas.2008389118)
Supplement: Supplementary File [file pnas.2008389118.sapp.pdf]

Personal Experiences Bridge Moral and Political Divides Better than Facts: Supporting Information

Emily Kubin, Curtis Puryear, Chelsea Schein, & Kurt Gray

Table of Contents

Sample Size and Demographics Across Studies 2

Procedure and Measure Description...3

Study 1: Lay Belief- Free Response...3
Study 2: Lay Belief- Multiple Choice...3
Study 3: YouTube Comments 4
Study 4: Testing the Model...5
Study 5: Field Study 7
Study 6: Specific Facts- Pilot Study...8
Study 6: Specific Facts...9
Study 7: Non-Relevant Experiences...10
Study 8: Non-Harmful Experiences 10
Study 9: Experience Gradient- Pilot Study 10
Study 9: Experience Gradient...10
Study 10: Real Experiences vs. Real Facts- Step 1 (Collecting Real Life Experiences)..............................11
Study 10: Real Experiences vs. Real Facts- Step 2 (Controlling for Harm) 11
Study 10: Real Experiences vs. Real Facts- Step 3 (Comparing Experiences and Facts)..............................11
Study 11: Real Op-ed Articles: Perceptions of Authors- Pilot Study 13
Study 11: Real Op-ed Articles: Perceptions of Authors..............................13
Study 12: News Transcripts..............................14
Study 13: Doubt and Moral vs Non-Moral Contexts 15
Study 14: Replication with Black Female Opponents: Pre-Test 16
Study 14: Replication with Black Female Opponents 19
Study 15: Perceptions of Scientists.............................. 19

Additional Analyses 21

Study 3: YouTube Comments 21
Study 4: Testing the Model...23
Study 5: Field Study 29
Study 6: Specific Facts- Pilot Study...32
Study 6: Specific Facts...33
Study 7: Non-Relevant Experiences...34
Study 8: Non-Harmful Experiences 36
Study 9: Experience Gradient- Pilot Study 38
Study 9: Experience Gradient...38
Study 10: Real Experiences vs. Real Facts- Step 2 (Controlling for Harm) 40
Study 10: Real Experiences vs. Real Facts- Step 3 (Comparing Experiences and Facts)..............................41
Study 11: Real Op-ed Articles: Perceptions of Authors- Pilot Study 43
Study 11: Real Op-ed Articles: Perceptions of Authors..............................43
Study 12: News Transcripts..............................45
Study 13: Doubt and Moral vs Non-Moral Contexts 46
Study 14: Replication with Black Female Opponents: Pre-Test 52
Study 14: Replication with Black Female Opponents 53
Study 15: Perceptions of Scientists.............................. 55

Links to Pre-Registrations 59

Additional BLM Study...60

Table S1

Sample sizes and demographics for all studies.

Study	Study Topic	Sample	Sample Size	Mean Age(SD)	Gender (% Female)	Ideology (% Liberal)
1	Lay Belief- Free Response	MTurk	251	35.41(11.92)	59.20%	41.43%
2	Lay Belief- Multiple Choice	Representative	859	36.12(12.17)	38%	32.1%
3	YouTube Comments	Archival	300,978*	-	-	-
4	Testing the Model	MTurk	177	35.41(10.68)	43.40%	49.80%
5	Field Study	Chapel Hill /Durham Residents	153	29.71(13.56)	51.00%	64.70%
6	Specific Facts	MTurk	194	37.51(10.76)	58.20%	46.90%
7	Non-Relevant Experiences	MTurk	273	34.82(12.59)	55.90%	50.80%
8	Non-Harmful Experiences	MTurk	255	35.85(12.45)	61.50%	51.40%
9	Experience Gradient	MTurk	408	34.38(11.58)	48.50%	46.20%
10	Real Experiences vs. Real Facts	Representative	1565	36.31(11.70)	50.60%	52.60%
11	Real Op-ed Articles: Perceptions of Author	MTurk	425	37.25(11.59)	44.30%	54.50%
12	News Transcripts	Archival	137*	-	-	-
13	Doubt and Moral vs Non-Moral Contexts	MTurk	508	36.18(12.60)	51.40%	47.00%
14	Replication with Black Female Opponents	MTurk	1897	39.66(12.81)	58.90%	51.4%
15	Perceptions of Scientists	Representative	1,411	41.74(15.43)	50.50%	54.00%

Note: Sample size indicates individuals included in analyses and not participants who failed attention checks. * represents the sample size for stimuli (Study 3, number of YouTube comments; Study 12, number of news transcripts).

Procedures and Measure Description for Study 1-15

Study 1: Lay Belief – Free Response

Participants completed a survey on MTurk where they were asked to: "Imagine someone disagrees with you on moral issues (e.g., same-sex marriage or abortion). What would make you respect their opinion?" Participants then responded to the following prompt, "I would respect their opposing opinion if it was based upon…."

Afterwards participants responded to basic demographic information. Age was measured through participants typing their age, in years in a text box. Participants then indicated their gender (1=male, 2=female), and their highest level of education (1=some high school, 2=high school degree or GED, 3=associates degree, 4=some college education, 5=Bachelor's Degree, 6=some graduate education, 7=Graduate Degree). Political ideology was measured through participants indicating their political leanings from, *very liberal* (1) to *very conservative* (7). Participants were finally thanked for their participation and received compensation. The demographic section was identical in all subsequent MTurk studies.

The qualitative data analysis was conducted in three steps. First, two research assistants blind to the hypothesis separately read all free responses, created categories, and then coded each response. Based on the research assistant generated categories, the first author then developed a final coding scheme. Finally, the same research assistants recoded the responses following the coding scheme. Raters agreed in 92.4% of the cases and the first author acted as referee for the remaining responses.

Study 2: Lay Belief- Multiple Choice

All participants first read a passage stating, "People use a variety of strategies when attempting to discuss topics with individuals who disagree with them. For example, some individuals may state statistics and facts while others share personal stories to attempt to make others understand their point of view. We are interested in seeing which strategy you think is best".

Afterwards participants responded to the following, "imagine an individual who holds a specific political stance based on *facts and data.* How much would you… 1) view this individual as rational, 2) respect this individual, 3) be willing to interact with this individual", using a 7-point scale from *not at all* (1) to *extremely* (7). Participants then responded to, "imagine an individual who holds a specific political stance based on *personal experiences.* How much would you… 1) view this individual as rational, 2) respect this individual, 3) be willing to interact with this individual", using the same 7-point scale. There was also an attention check below these items that asked participants to select "somewhat"—the midpoint on the 7-point scale. Afterwards participants completed the demographic measures, were thanked for completing the study, and were paid for participation.

Study 3: YouTube Comments

We used the tuber package in R studio and the YouTube API to collect meta-data (e.g., video titles, view counts, reply counts etc.) for the top 100 results of six search terms related to abortion. We used "abortion statistics," "abortion evidence," "abortion facts," to target videos making argument based on statistics and facts. The terms "abortion experience," "abortion story," and "abortion survivor" targeted videos with personal experiences. We then excluded videos that had fewer than 100 comment, were not about abortion, or were duplicates—resulting in a corpus of 194 videos.

Four raters independently coded videos as primarily containing personal experiences, facts, or other. Videos containing a mix of experience and facts (e.g., news segments containing both statistics and testimonials) were coded as other. Common examples of experience videos included people telling stories about their abortion on a webcam and news interviews with women who had abortions. Informational videos from websites like *Buzzfeed* and *NowThis* were common examples of videos coded as facts. Pairs of raters agreed on 81% of cases overall, yielding Cohen's Kappa's of .65, .66, and 1.00 (agreement was perfect for one pair of coders who only coded results for the "abortion survivor" term, which consisted almost entirely of videos that clearly focused on personal experiences). After resolving discrepancies, our corpus contained 67 videos coded as facts, 51 as experience, and 76 as neither.

We then collected the total 300,978 top-level, user comments (i.e., comments that were not replies to other comments) across all 194 videos[1]. We focused on top-level comments because they respond directly to the videos, rather than to other users. Thus, they should better capture people's reactions to the content of the videos, which we manually coded to ensure they reflect personal experiences, facts, or other. All our confirmatory predictions and analyses reported here focus on top-level comments. The data set with the additional 631,413 replies is available at https://osf.io/j4czu/. All comments were stripped of URLs and emoticons prior to analysis.

Text Analysis. We used LIWC2015 (*1*) to estimate the tone of each comment. LIWC measures psychological processes by counting the frequency of words from validated dictionaries. For example, LIWC's anger dictionary contains 230 anger-related words and stems (e.g., hate, kill, annoyed). LIWC outputs anger scores by calculating the percentage of words in a given text that appear in the anger dictionary. Some of LIWC's dictionaries are arranged hierarchically. The anger and sadness dictionaries, for instance, are subsets of the negative emotion dictionary. Each occurrence of an anger or sadness word also increments the negative emotion score.

Dependent variables. We analyzed eight different outcomes generated by LIWC. Three of these—tone, authenticity, and analytic language—were summary variables introduced in the 2015 version of LIWC. LIWC rescales summary variable to range from 0 to 100. All other

[1] We originally analyzed a subset of our corpus of comments before pre-registering confirmatory hypotheses on the remaining data. Because results were consistent across the exploratory and confirmatory data sets, we report results for the combined data here. Results for each subset of data can be found at https://osf.io/j4czu/

variables represent the percentage of words in a comment that occur in the respective dictionary (e.g., a comment with 10 total words that contains both the words "hate" and "kill" will receive an anger score of 2.5). Dependent variables can be found in Table S2:

Table S2

Description of Dependent Variables

Emotional tone – higher scores are associated with more positivity. Lower numbers reveal more anxiety, sadness, and hostility. Scores at the midpoint suggest emotional ambivalence.

Authentic – higher scores correspond with more honest and personal text. Lower scores signify more guarded and distanced language.

Analytic – higher scores are associated with more formal, logical thinking. Lower numbers represent more personal, in the moment, and narrative types of thinking.

Positive emotion words (e.g., love, nice, sweet)

Negative emotion words (e.g., hurt, ugly, nasty)

Anger words (e.g., hate, kill, annoyed)

Sadness words (e.g., crying, grief, sad)

Affiliation drive words (e.g., ally, friend, social)

Study 4: Testing the Model

At the beginning of the study participants reported their stances on a variety of political issues (i.e., taxes, coal mining, and gun policy). Participants responding to the following prompts; "Do you think there should be more taxes on businesses in the United States", "Do you think there should be more coal regulations (i.e., restricting coal practices) in the United States", and "Do you think there should be more gun regulations (i.e., restricting access to guns) in the United States?"**,** using a 6-point scale from *strongly disagree* (1) to *strongly agree* (6).

Participants then read about three people who disagreed with them on these three political issues either based on 1) factual knowledge or 2) personal experiences (i.e., our epistemological conditions). The three vignettes each participant read were the same epistemological condition (e.g., all three opponents based their stances in their factual knowledge). After reading each vignette, participants responded to manipulation checks and the dependent variables. See Table S3 for descriptions of the vignettes.

Table S3

Vignettes used in Study 4

Vignette Topic	Condition	Vignette
Tax Policy	Personal Experience	Samantha supports [raised/reduced] taxes for businesses because she had first-hand experience with business taxes and is facing bankruptcy due to recent [household tax increases/tax increases to her business.
	Fact	Samantha has not had any experience with business taxes, but she supports [raised taxes/reducing taxes] for businesses based on facts she learned while reading extensively about the topic.
Coal Policy	Personal Experience	Bill supports [less/more] restrictions on coal mining because he has first-hand experience with [mining and lost his job to new coal regulations/ the mining industry after it left his water supply unsafe to drink].
	Fact	Bill has not had any experience with coal mining, but he supports [less restrictions on coal mining/more restrictions on the coal industry] based on facts he learned while reading extensively about the topic.
Gun Policy	Personal Experience	Dylan supports [gun regulations (i.e., restricting access to guns)/gun rights (i.e., access to guns)] because he had first-hand experience with guns [when his daughter was hit by a stray bullet/ when he shot an intruder to protect his young daughter].
	Fact	Dylan has not had any personal experience with guns, but he supports [gun regulations (i.e., restricting access to guns)/ gun rights (i.e., access to guns) based on facts he learned while reading extensively about the topic.

Measures. To assess the extent to which our conditions captured personal experience and factual knowledge, we developed manipulation checks that were used in all subsequent studies. To measure personal experience, participants reported their level of agreement with the following items, “this person has personal involvement with the topic”, “this person has first-hand knowledge of this issue”, and “this person has an authentic experience with this issue”, using a 7-point scale from *strongly disagree* (1) to *strongly agree* (7). To measure factual knowledge, participants reported their level of agreement with the following items, “this person assessed data before coming to their viewpoint”, “this person read extensively about the topic before coming to this stance”, and “a knowledge of relevant statistics helped this person come to this conclusion”, using a 7-point scale from *strongly disagree* (1) to *strongly agree* (7).

After responding to the manipulation checks, participants reported the extent to which they viewed the character as rational, respected them, and were willing to interact with them. In all subsequent studies (unless otherwise noted), the measurement of the variables was identical.
To measure rationality, participants indicated how much they thought the individual, “is rational for holding their stance”, “has a stance that makes sense”, “is logical for holding their stance”, using a 7-point scale from *not at all* (1) to *extremely* (7). Respect was measured by participants indicated how willing they would be to, “respect this person’s viewpoint”, “be considerate of this person’s stances”, and “take this person’s point of view”, using a 7-point scale from *very unwilling* (1) to *very willing* (7). Finally, participants indicated their willingness to interact with the opponent by indicating how willing they would be to, “have a general discussion with this person”, “interact with this person”, and “exchange ideas with this person”, using a 7-point scale from *very unwilling* (1) to *very willing* (7). Afterwards, participants reported basic demographics (i.e., age, gender, political ideology, and education). They were then thanked for participation and received compensation.

Study 5: Field Study

After being recruited, participants completed a demographic form which included basic demographic questions as well as the following question, “Do you think there should be more gun regulations (i.e., restricting access to guns) in the United States?” Participants responded using a 6-point scale from *strongly disagree* (1) to *strongly agree* (7). The researcher would then tell the participant they would be speaking with another participant (i.e., the confederate) who disagreed with their stance on guns. The participant would then be introduced to the confederate and be asked to state why they hold their stance on gun policy. Based on the participant’s stance on guns, and the condition (i.e., facts vs. personal experiences) the participant was randomly assigned to, the confederate would say a specific script[2].. Details about these scripts can be found in Table S4. To increase the potential for incivility, the confederate also suggested the participant was unamerican for holding their view.

After the confederate made their statement, the participant had an opportunity to respond to the confederate. The researcher then ended the experiment and the participant was given a debriefing form, which explained the true nature of the study. The confederate then rated the participant on the following items, how rational the participant seemed to view their stance, how much the participant seemed to respect them, and how willing the participant was to interact with them using a 7-point scale from *not at all* (1) to *extremely.* Since confederates were aware of the condition when coding and were aware of the hypotheses, the dialogues were also recorded so other research assistants—blind to the hypothesis—would be able to recode the interactions for these items.

[2] The factual knowledge vignette in Studies 5-7, 10 and 14 did not highlight lack of personal experience to assure the presence versus absence of personal experience were what explained these effects.

Table S4

Scripts used in Study 5

Condition	Script
Personal Experience	Well, I think you're wrong about gun policy. I am [for/against] gun control, and I feel pretty strongly about that. I don't know how you can call yourself an American if you don't think that guns [hurt people and communities/ keep our communities safe]. I believe this because [my mom was hospitalized after being hit by a stray bullet/ was able to protect herself with her gun when someone attacked her], so my personal experience has really made me feel strongly about this.
Fact	Well, I do think you're wrong about gun policy. I am [for/against] gun control, and I feel pretty strongly about that. I don't know how you can call yourself an American if you don't think that guns [hurt people and communities/keep our communities safe]. I believe this because I have read many books and governmental reports on gun policy, so my factual knowledge has really made me feel strongly about this. .

Blind To Hypothesis RA Codes. After collection of the data was complete, another set of research assistants listened to the recordings of the dialogue. This allowed the research assistants' coding to not be swayed by condition. The research assistants independently coded each of the recordings. Coders reported the extent to which the participant seemed to view the confederate as rational from *not at all* (1) to *extremely* (7). Further, coders rated the extent to which participants seemed to respect the confederate and the extent to which they seemed willing to interact with the confederate using a 7-point scale from *very unwilling* (1) to *very willing* (7). These scales were based on the previously used scale items used in Study 4.

Study 6: Specific Facts- Pilot Study

A pilot test was completed to assure the new factual knowledge prompts had high degrees of specificity. Participants read a variety of sentences and then rated the extent to which each was; concrete, abstract, vague, specific, and detailed using a 7-point scale from *strongly disagree* (1) to *strongly agree* (7). We predicted our proposed factually specific sentences (i.e., "someone reads in an annual federal report that 73% of murder in the United States are committed with firearms"; "Someone reads in an annual federal report that civilians use guns to defend themselves over 989,000 times per year"), would be rated as very specific. These facts were retrieved from: https://www.justfacts.com/guncontrol.asp. Participants responded to our personal experience items used in previous studies, (i.e., "someone shoots an intruder to protect his young daughter", and "someone's. young daughter is hit by a stray bullet"). Participants also responded to a variety of control sentences (e.g., "someone finds something"). Table S5 indicates the

factual items were viewed as highly specific—suggesting these prompts would be appropriate for testing the extent to which specificity could impact rationality and respect of adversaries.

Table S5

Mean ratings of specific for a variety of sentences in the pilot study of Study 6.

Prompts	Mean Perceived Specificity(SD)
Someone reads in an annual report that 73% of. Murders in the United States are committed with firearms†	5.12(1.05)
Someone shoots an intruder to protect his young daughter**	4.52(1.03)
Someone's young daughter is hit by a stray bullet**	4.38(1.03)
Someone reads in an annual federal report that civilians use guns to defend themselves over 989,000 times per year†	4.88(1.23)
Someone completes their annual tax return early	4.32(.99)
Someone leaps from a moving car	4.05(1.17)
Someone finds something	2.38(1.17)
Someone watches the Jeopardy gameshow at 7:30pm every Tuesday	5.08(1.22)
Someone calculates gas mileage using a Casio calculator	4.83(1.00)
Someone moves	2.53(1.12)

Note: †= Specific Factual items, **= Personal Experience items

Study 6: Specific Facts

Participants reported their stance on guns and then read a vignette about an individual who disagreed with their stance on guns because of 1) personal experience, (these vignettes were used in previous studies) or because of 2) *specific* factual knowledge. In this condition participants read, "Tyler recently read in an annual federal report that [civilians use guns to defend themselves over 989,000 time per year/ 73% of murders in the United States are committed with firearms]. Tyler supports gun rights (i.e., access to guns)". Participants then rated the opponent on experience and factual knowledge (our manipulation checks), and rationality, respect, and willingness to interact measures from Study 4.

Study 7: Non-Relevant Experiences
Participants reported their stance on guns and then were randomly assigned to read about an individual who disagreed with them on gun policy who had 1) relevant personal experience with guns (e.g., protected their family from an intruder with a gun), 2) non-relevant personal experience (i.e., had their business go into bankruptcy), or 3) have factual knowledge about guns (i.e., recently read facts in the American Gun Policy Report). Participants then responded to the manipulation checks as well as the rationality, respect, and willingness to interact items.

Study 8: Non-Harmful Experiences
After reporting their stance on gun policy, participants were randomly assigned to read about an individual who disagreed with them on gun policy who had 1) harmful personal experience with guns (e.g., protected their family from an intruder with a gun), 2) non-harmful personal experience with guns (i.e., took a firearm safety course), or 3) have factual knowledge about guns. Participants then responded to the manipulation checks as well as the rationality, respect, and willingness to interact items.

Study 9: Experience Gradient – Pilot Test
Participants used the Inclusion of Other in the Self Scale (IOS; *2*) to rate the extent to which specific relationships are related to one another. In a randomized order, individuals reported the extent to which 1) The Self and The Self, 2) The Self and their brother, 3) The Self and their friend, 4) The Self and an acquaintance living in another state, and 5) The Self and somebody they read about in a book, were related to one another. Afterwards participants reported demographic information and were paid for participation.

Study 9: Experience Gradient
After reporting their stance on gun policy, participants were randomly assigned to read about one individual who disagreed with their stance on guns. The personal experience gradient items were based on the pilot study. Participants were randomly assigned to read about; the self, their brother, their friend, an acquaintance living in another state, or somebody they read about in a book.

The vignette was written as such, “Tyler [supports gun regulations (i.e., restricting access to guns)/is against gun regulations (i.e., access to guns)] because [personal experience gradient item (e.g., because his brother)] has first-hand experience with guns when he [was hit bit a stray bullet/shot an intruder to protect his family]”. Afterwards participants reported their perceptions of Tyler’s personal experience, factual knowledge, rationality, their willingness to respect him, and their willingness to interact with him, as well as the demographic items.

Study 10: Real Experiences vs. Real Facts- Step 1 (Collecting Real Life Experiences)
We first collected real life personal experiences with guns from MTurk workers. While we initially collected personal experiences from all 3 topics (as reported in our pre-registration) we decided to use only personal experiences with guns in subsequent parts of this study. This was because the pro-immigration personal experiences were substantially more compelling than the anti-immigration experience. Pro and anti-abortion personal experiences were similarly unbalanced. The subsequent explanation of our methods will therefore only focus on participants who chose to write about gun policy.

Participants first reported their stance on gun policy using a 7-point scale from *strongly pro-gun* (1), to *strongly anti-gun* (7). Afterwards participants reported their moral conviction about gun policy by responding to 2 moral conviction items (*3*) using a 6-point scale from *not at all* (1) to *very much* (7). The items were, "To what extent is your position on gun policy deeply connected to your beliefs about fundamental right and wrong?", and "To what extent is your position on gun policy a reflection of your core moral beliefs and convictions". These items were then averaged together, and only those individuals who scored above a 3 (the mid-point) on the moral conviction items had their experiences included in later parts of this study.

Study 10: Real Experiences vs. Real Facts- Step 2 (Controlling for Harm)

Participants were randomly assigned to read 8 of the real personal experiences[3]. Participants read both pro-gun and anti-gun personal experiences regardless of their own stance on gun policy. After reading each personal experience, participants responded to a 3-item compellingness measures using a 7-point Likert scale from *strongly disagree* (1) to *strongly agree* (7). The items were, "The passage was powerful", "The passage seemed compelling", "I found the passage impactful", α= .96. Participants then responded to a 3-item harm measure using the same 7-point Likert scale. The items were, "The passage involves suffering", "The passage highlights victimization", and "This passage focuses on harm", α=.89.

Study 10: Real Experiences vs. Real Facts- Step 3 (Comparing Experiences and Facts)

After controlling for harm within the experiences by choosing pro- and anti-gun personal experiences with similar levels of harm (i.e., low, medium, and high), we then compared these real experiences to well-regarded facts by pro and anti-gun advocates. Three statistics were collected from both the National Rifle Association's and Every Town for Gun Safety's website. Both of these organizations are the leaders in pro and anti-gun advocacy in the Unites States, respectively—thus we believe the statistics they use on their websites are viewed as most convincing by gun policy advocates. Thus, we chose facts that we believed had the best chance of increasing rationality and respect for opponents who share such statistics. See Table S6. for the statistics used in Study 10 Step 3.

Participants first reported their stance on guns by using the same measure as previous studies. They were then told they will read about somebody who disagrees with their stance on gun

[3] There were 49 personal experiences collected that met our pre-set inclusion criteria—but only 47 were included in this survey due to technical errors with the survey.

regulations due to either 1) reading real data and statistics online about guns or 2) having real personal experiences with guns. Participants were then randomly assigned to read about an opponent who read one of the 3 facts, or had one of the 3 personal experiences (either low, medium, or high levels of harm). After reading one of these vignettes, they responded to our manipulation checks, and all of our dependent variables (i.e., doubt, rationality, respect, wiliness to interact).

Table S6

Vignettes used in factual condition for Study 10 Step 3

Participant Gun Stance	Vignette
Pro-Gun	1. "I am anti-gun because I read a statistic that every year nearly 2,900 children and teens are shot and killed and nearly 15,600 more are shot and injured."
	2."I am anti-gun because I read a statistic that half of all Americans still live in states where a convicted felon, domestic abuser, or fugitive can skip a background check by finding an unlicensed seller at a gun show or online."
	3."I am anti-gun because I read a statistic that Americans are 25 times more likely to be murdered with a gun than people in other developed countries."
Anti-Gun	1."I am pro-gun because I read a statistic that murder rates were 19.3% higher when the Federal assault weapon ban was in effect."
	2."I am pro-gun because I read a statistic that total violent crime has fallen to a 44 year-low and murder to an all-time low, while ownership of the firearms and magazines that gun control supporters want banned has risen to all-time highs.”
	3."I am pro-gun because I read a statistic that 40 percent of felons had not committed one or more crimes because they feared that their prospective victims were armed. Thirty-four percent had been scared of, shot at, wounded, or captured by an armed victim."

Study 11: Real Op-ed Articles: Perceptions of Authors- Pilot Study

To ensure the selected op-eds indeed highlighted personal experience (or factual knowledge), a within subject pre-test was completed. Opinion pieces from the New York Times were selected by entering into the google.com search bar: "New York Times opinion guns". This search provided many results from articles both supporting gun rights, and promoting gun safety. Four articles were selected, two highlighted personal experiences with guns, one of these had a pro-gun stance, the other an anti-gun stance. The other two articles highlighted data and statistics about guns, one of these had a pro-gun stance, the other an anti-gun stance. These opinion pieces had comparable lengths to one another.

The article with a pro-gun stance based on facts was written by the president of the Crime Prevention Research Center, who highlighted that over 91% of law enforcement officials support concealed carrying of guns, whereas the pro-gun writer with personal experience, highlighted a time a man entered her childhood home and her mother confronted him with a rifle. The article with an anti-gun stance based on facts was written by a columnist who highlighted statistics such as, "between 8.5 million and 15 million assault rifles are in circulation", whereas the anti-gun experiential op-ed was written by a survivor of a school shooting about her experience that day.

Participants were randomly assigned to read two real opinion pieces from the New York Times[4]. Participants were shown both a personal experience and factual knowledge article (though either article could be a pro-gun or anti-gun op-ed). After reading each article, participants then reported the extent to which the article was based in personal experiences, from *strongly disagree* (1) to *strongly agree* (7), (example item: "the writer has personal involvement with the topic). Participants also rated the extent to which each article was based in factual knowledge using the same 7-point scale, (example item: "the writer assessed data before coming to their viewpoint").

Study 11: Real Op-ed Articles: Perceptions of Authors
Participants reported their stance on gun policy and then read a New York Times opinion piece written by a person with an opposing stance on gun policy. This article was either based in factual knowledge, or personal experience (as supported by ratings from the pilot study). Participants then rated the extent to which the writer seemed rational, how willing they were to respect the writer, and how willing they were to interact with the writer, using the same measures as in study 4.

Study 12: News Transcripts

---

[4] The articles can be found at the following links:
Pro-gun Facts: https://www.nytimes.com/2018/02/12/opinion/politics/background-checks-gun-violence.html
Pro-gun Experience: https://www.nytimes.com/2018/03/05/opinion/mom-gun-safety-intruder.html
Anti-gun Facts: https://www.nytimes.com/2018/02/25/opinion/america-is-the-gun.html
Anti-gun Experience: https://www.nytimes.com/2018/02/18/opinion/florida-school-shooting-guns.html

Transcripts of interviews from Fox News and CNN were collected between the following time ranges (2002-2004; 2008-2010; 2015-2017). All transcripts involved an interviewer speaking with an individual from the opposing side (i.e., Fox interviewers speaking with liberals and CNN interviewers speaking with conservatives).

Research assistants gathered 137 transcripts and then coded for a variety of measures using a 7-point scale 1=*not at all present* 7=*extremely present*. Four research assistants coded the extent to which factual knowledge and personal experiences were discussed by the interviewee. Two research assistants coded the first half of the transcripts, and the other two coded the second half. Afterward, the transcripts were edited so only the interviewer's words were left in the transcript. The research assistants switched transcripts with one another (thus the second round of coding was with the other half of the transcripts) and coded the following items; 1) how rational the interviewer believes the interviewee is 2) how respectful the interviewer is of the interviewee 3) how willing the interviewer is to interact with the interviewee. Research assistants were unaware of which show and which timeframe the transcripts were from during the second round of coding. Of the 145 transcripts, 32 were from the 2002-2004 timeframe, 72 were from 2008-2010, and 41 were from 2015-2017. Seventy of the transcripts were from Fox News shows and 75 were from CNN News shows. See Table S7 for examples of excerpts of transcripts that highlighted experience vs. facts.

Table S7

Examples of high experiential and factual quotes from news transcripts in Study 12.

Quotes from Interviewees Highlighting Experience	Quotes from Interviewees Highlighting Facts
"Well, I feel like what's really important right now is absolutely jobs and the economy. I mean, this is something that transcends party lines, and I believe that we're really stuck today on social issues. And I think at the end of the day, those things are not going to be as important as jobs. I can speak from personal experience myself. My husband has been unemployed. He is a very smart educated man. And the jobs just aren't there. We are true middle class and the middle class is suffering. Education is another thing. My daughter is going to be graduating from high school and we are now looking at colleges."<br><br>**Experiential Rating for Interview: 6.5**	"We've seen the unemployment rate fall from its peak of 6.3 percent now down to 5.7. We've seen about 250,000 more jobs created since the summer. That's not enough. And we want to see more job creation and want things to get better for the American worker. And we think we have policies in place to do that, and we certainly are working hard on that goal."<br><br>**Factual Rating for Interview: 7**
"And I can tell you, from constituents in my district, who I see all of the time, they are dying the most hideous deaths -- rare blood disorders, cancers, pulverized glass in their lungs. It's absolutely horrible, what they're going through"<br><br>**Experiential Rating for Interview: 6.5**	"The system was set up in such a way that the payroll tax, the 6.2 percent that we now pay, and matched by the employer, fully funds, as your sound bites indicated, fully funds Social Security, but not now. The 2 percent reduction, that is from 6.2 to 4.2 percent, that actually is backfilled by the general fund. And that is all borrowed money. Now, the politics of it, one year from now, that 2 percent is added back in, a 48 percent reduction -- excuse me -- a 48 percent increase."<br>**Factual Rating for Interview: 7**

Study 13: Doubt and Moral vs. Non-moral Contexts

Participants in the moral condition reported their stances on taxes, coal mining, and gun policy with the same 6-point scale used in previous studies. Those in the non-moral condition were told a little bit about risks of investments, purchasing a diesel car, and different kinds of blenders. They then used the 6-point scale to respond to the following prompts regarding their stances on these non-moral topics, "I prefer investing in a start-up company", "I prefer a diesel car", and "I prefer the Vitamix Blender". Participants read about 3 individuals in short vignettes who either agreed or disagreed with their own stances (and held this stance based on facts or personal experience). Those in the moral condition read identical vignettes to those in Study 4. Participants in the non-moral condition read the vignettes in Table S8.

Though participants read three vignettes, all manipulations were between subjects (e.g., participants only read vignettes that agreed with them or only vignettes that disagreed with them). Participants then responded to the manipulation checks, a measure focusing on the extent to which participant's doubted opponents, and the rational, respect, and willingness to interact items. Afterwards participants responded to demographic questions.

Table S8

Non-moral vignettes used in Study 13

Vignette Topic	Condition	Vignette
Investments	Personal Experience	Samantha supports investing in [large/start-up] companies because she had first-hand experience with investing in [start-up/large] companies and has had negative experiences with these investments.
	Fact	Samantha has not had any experience with investing, but she supports investing in [large/start-up] companies based on data from a governmental agency.
Car Choice	Personal Experience	Bill supports selecting a [gasoline/diesel] car because he had first-hand experience with purchasing cars and had negative experiences with [diesel/gasoline] cars.
	Fact	Bill has not had any experience with purchasing cars, but he supports selecting a [gasoline car/diesel] based on statistics he heard on the news.
Blender Choice	Personal Experience	Dylan prefers the [NutriBullet/Vitamix] Blender because he had first-hand experience with purchasing blenders and had negative experiences with the [Vitamix/NutriBullet] Blender.
	Fact	Dylan has not had any experience with kitchenware, but he prefers the [NutriBullet/Vitamix] Blender based on data from an annual consumer report.

Study 14: Replication with Black Female Opponents: Pre-Test

To pre-test the items we recruited 192 participants from MTurk. Participants read a subset of the 6 vignettes, they either read all the pro-gun rights vignettes, or all the pro-gun restriction vignettes. Which set they read was randomized and not based on whether they supported either movement. Participants read about a Black woman who based her stance on personal experiences, facts, or provided no rationale (control condition). The vignettes can be found in Table S9.

After reading each vignette (in a randomized order), participants reported the extent to which each vignette highlighted several dimensions (i.e., how, specific, evocative, and salient each vignette was). All dimensions used a 7-point scale from *strongly disagree* (1) to *strongly agree* (7). The items used for each dimension can be found in Table S10.

Analyses indicated that the factual vignettes were rated has higher on all three dimensions, which in theory "stacks the cards" further against our favor (i.e., making it less like personal experiences would be rated as more rational or incite more respect). See Table S44 and S45 for mean ratings for each vignette across dimensions.

Table S9

Vignettes used in Study 4

Condition	Vignette
Personal Experience	Tia Williams, a Black American, supports gun [control/rights] (i.e., [restricting/] access to guns). When she talks to people about it, she tells them that it is because she [and her young daughter were hit by stray bullets/had to shoot an intruder to protect herself and her young daughter].
Fact	Michelle Brown, a Black American, supports gun [control/rights] (i.e.,[restricting/] access to guns). When she talks to people about it, she tells them that it is because [73% of murders in the United States are committed with firearms[5] and gun-related deaths are now the third leading cause of death of American children[6]/each year, over 989,000 civilians use guns to defend themselves[7], including 200,000 women who use guns to defend themselves against sexual violence[8]].
No Rationale	Cassandra Baker, a Black American, supports [control/rights] (i.e., [restricting/] access to guns).

[5] Fact from the Every Town for Gun Safety Website

[6] Fact from **https://www.dosomething.org/us/facts/11-facts-about-guns**

[7] Fact from National Rifle Association website

[8] Fact from **https://americangunfacts.com/**

Table S10

Variables and reliability of items used to assess comparability of vignettes in Study 14 pre-test

Variable	Items
Specificity	1."The statement is… concrete”<br>2." The statement is… abstract”<br>3." The statement is… vague”<br>4." The statement is… specific”<br>5." The statement is… detailed”
Evocative	1." The statement … is detailed and descriptive”<br>2." The statement… captures my attention”<br>3." The statement … has vivid language”
Salient	1." The statement is … related to an important discussion<br>in the United States”<br>2." The statement is… relevant to society”<br>3." The statement is … meaningful for our understanding<br>of social and political issues in this country”

Study 14: Replication with Black Female Opponents

For the full study, participants reported their stance on gun policy in the United States using our standard gun stance question used in previous studies. Participants were then randomly assigned to read about an opponent who disagreed with them based personal experiences, facts, or provided no rationale. Participants then responded to our standard manipulation check and rationality, respect, interact measures.

Study 15: Perceptions of Scientists

Participants first reported their stance on immigration by responding to, "The United States should increase the number of immigrants deported from the country each year", using a 6-point scale from *strongly disagree* (1) to *strongly agree* (6). Afterwards, participants were randomly assigned to read about somebody who disagreed with them on immigration based on factual knowledge, personal experience, or who was a scientist conducting research on the topic.

The factual knowledge and personal experience conditions were piloted to test for specificity. The factual knowledge condition was viewed as more specific—we then adapted the factual condition into the scientist condition by stating the statistic was discovered by the political opponent as part of grant funded research. See Table S10 for vignettes used in this study.

Participants responded to our standard manipulation checks, doubt, rationality, respect and willingness to interact measures. They also completed the top 3 factor loadings (α=.83) from the Belief in Science Scale (*4*). This was 3 items using a 6-point scale from *strongly disagree* (1) to *strongly agree* (7). Items were, "the scientific method is the only reliable path to knowledge", "the only kind of knowledge we can have is scientific knowledge", "science is the most efficient means of attaining truth.

Table S11

Vignettes used in Study 15

Condition	Vignette
Experience	Rodrigo supports [decreasing/increasing] the deportation of illegal immigrants based on his first-hand experience: His father was [deported and later assaulted by a local street gang/assaulted and severely injured by a gang of illegal immigrants] and Rodrigo had to pay for the medical expenses.
Fact	Rodrigo supports [decreasing/increasing] the deportation of illegal immigrants based on data and statistics he read online. In a report titled “Immigration - Related Crime”, published by the Immigration Policy Think Tank, Rodrigo read that there has been a 45% increase since 2016 in incidences of [deportees being assaulted by local street gangs/people being assaulted by gangs of illegal immigrants].
Scientist	Rodrigo is a scientist who supports [decreasing/increasing] the deportation of illegal immigrants based on his research. He received a grant from the National Science Foundation to examine immigration-related crime. His analysis—published in “Immigration Policy”—found a 45% increase since 2016 in incidences of [deportees being assaulted by local street gangs/people being assaulted by gangs of illegal immigrants]

Additional Analyses

Study 3: YouTube Comments

Experience videos generated comments with significantly more positive tone than videos containing facts or other videos about abortion (see Tables S12 and S13 for model results and descriptive statistics). Increased use of positive emotion and affiliation words largely drove the relative positivity of comments on experience videos. Use of negative emotion words did not differ across video type overall. However, experience videos produced slightly more sad words and marginally fewer anger words. Unsurprisingly, comments responding to videos about facts used much more analytic language than comments on experience videos. Comments did not differ in authenticity across video type.

We conducted two secondary analyses. To explore how comment sentiment was distributed across videos, we calculated the average score on all eight outcome variables for each video. Figure S1 depicts how emotional tone scores were distributed across videos sorted by type. We then treated each video as a case in ANOVAs, which generated nearly identical results as the multi-level models (see Table S13 for results from Tukey tests). Lastly, as a robustness check, we re-ran all the multi-level models after deleting multiple comments from the same user. This reduced the data set to 224,389 comments, all from unique users. Again, results were nearly identical to our models using the full data. The slightly more frequent use of anger words in comments responding to fact (relative to experience) videos became significant ($p = .02$), and the significant effect upon sad words became marginal ($p = .07$)

These results suggest that YouTube videos sharing personal experience with abortion attract more positive comments than videos sharing facts. All other videos about abortion in our data set produced comments on the negative end of LIWC's tone scale. For discussion about abortion on YouTube, negativity is the norm. However, citing personal experiences produces comments with substantially more positivity.

Table S12

Study 3 multi-level model results

	Experience vs. Facts		Experience vs. Other		Random Effects	
Outcome	Estimate(*SE*)	*p*	Estimate(*SE*)	*P*	Video ID Intercept *SD*	Video ID Residual *SD*
Tone	-10.55(1.90)	<.001	-8.46 (1.85)	<.001	9.80	35.80
Positive Emotion	-3.20(.42)	<.001	-2.97(.41)	<.001	2.14	8.39
Affiliation	-.89(.15)	<.001	-.77(.15)	<.001	.76	3.84
Negative Emotion	.11(.40)	.775	-.28(.39)	.471	2.01	9.59
Sad	-.24(.11)	.037	-.19(.11)	.080	.54	3.51
Anger	.42(.25)	.092	.22(.24)	.363	1.20	7.19
Authentic	1.81(1.17)	.125	.78(1.14)	.490	5.76	33.36
Analytic	8.43(1.34)	<.001	10.27(1.31)	<.001	6.71	33.48

Table S13

Descriptive statistics and Tukey tests for YouTube comments

Outcome	Experience Mean(*SD*)	Facts Mean(*SD*)	Other Mean(*SD*)
Tone	43.77(15.35)^{a}	33.08(6.77)^{b}	35.28(8.36)^{b}
Positive Emotion	7.05(3.69)^{a}	3.81(1.07)^{b}	4.03(1.70)^{b}
Affiliation	2.25(1.27)^{a}	1.34(0.54)^{b}	1.48(0.65)^{b}
Negative Emotion	5.15(2.62)^{a}	5.28(2.07)^{a}	4.88(1.84)^{a}
Sad	0.80(0.44)^{a}	0.56(0.44)^{a}	0.59(0.72)^{a}
Anger	2.38(1.75)^{a}	2.80(1.27)a	2.59(1.17)^{a}
Authentic	27.82(7.61)^{a}	29.64(4.74)^{a}	28.48(7.06)^{a}
Analytic	40.78(8.12)^{a}	49.21(6.34)^{b}	51.12(7.25)^{b}

Note: means without a superscript letter in common are significantly different from one another at $p < .05$.

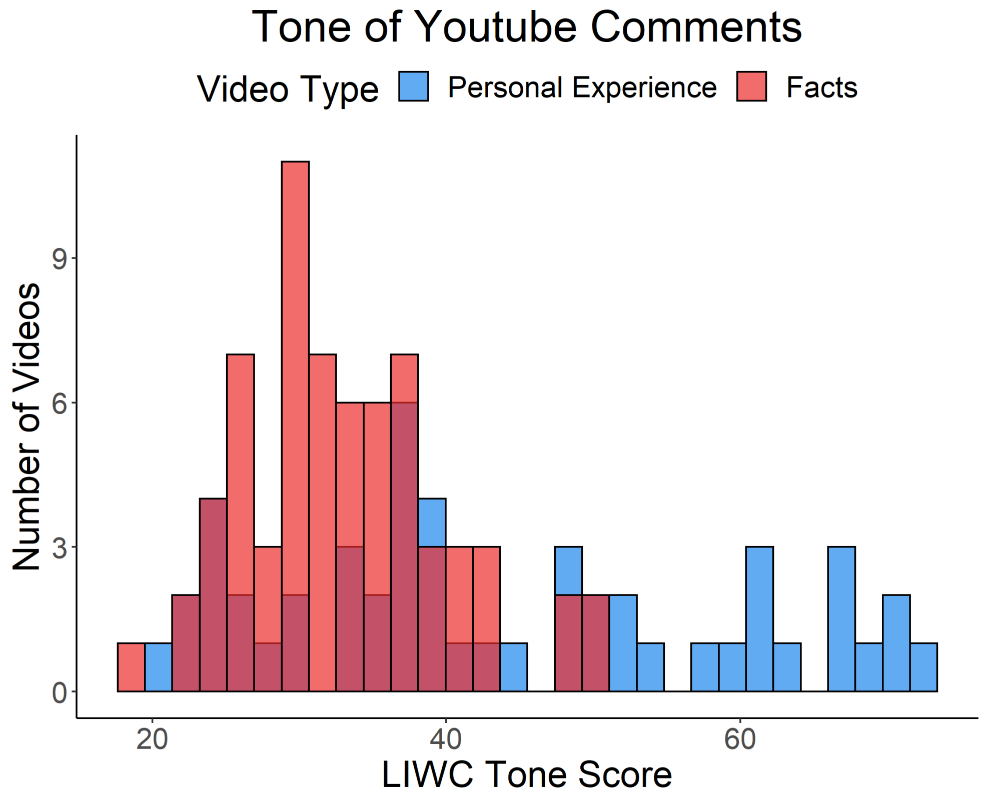

Figure S1. Histogram of emotional tone scores (i.e., the average LIWC tone score for all comments on a video) for the 51 experience and 67 facts/evidence videos. LIWC estimates tone as the difference between the proportion of positive and negative emotion words in a document, then rescales scores to range from 0-100. Higher scores indicate more positive tone.

Study 4: Testing the Model

Manipulation Check: Collapsing across stories, the experiential vignettes were viewed as having more personal experience and the factual vignettes were viewed as having more factual knowledge, suggesting our manipulation was successful. See Table S14.

Table S14

Means for manipulation check measures in Study 4

	Facts Means (SD)	Personal Experience Means (SD)	Inferential	Cohen's d
Personal Experience	2.42(1.41)	5.77(.85)	$t(175)=-18.94$, $p<.001$	2.88
Facts	5.06(1.21)	4.20(1.36)	$t(175)=4.46$, $p<.001$	.67

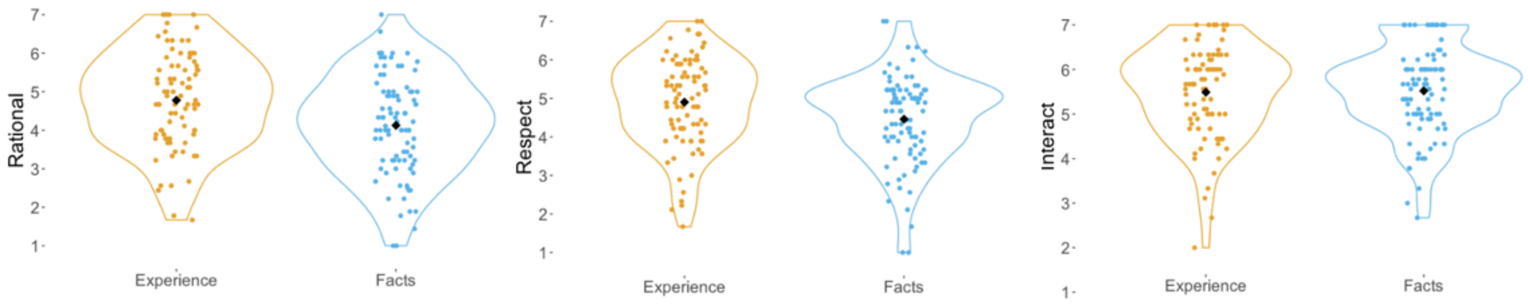

Figure S2. Violin plots of the density of ratings around the mean for Study 4 for the dependent variables. The black diamond indicates the mean score.

Table S15

PROCESS Macro mediational analyses in Study 4[9].

Outcome Variable	Mediator(s)	Indirect effect of Mediator(s) (SE), [95% CI]	Effect of Condition on Mediator (SE), [95% CI]	Effect of M1 on M2 (SE), [95% CI]	Effect of Mediator on DV (SE), [95% CI]	Total Effect (SE), [95% CI]	Direct Effect (SE), [95% CI]
Respect	Rationality	B=.48(.14), [.21, .78]	B=.65(.19), [.27, 1.03]		B=.74(.04), [.66, .82]	B=.44(.18), [.10, .79]	B=-.04(.11), [-.25, .11]
Interact	Rationality (M1) Respect (M2)	B=.17(.07), [.05, .33]	B=.65(.19), [.27, 1.03]	B=.74(.04), [.66, .82]	B=.34(.10), [.15, .54]	B=-.03(.15), [-.33, .27]	B=-.24(.14), [-.51, .03]

[9] Studies 4 and 13 had a within subject design, therefore we also ran multilevel analyses for these studies.

Table S16

PROCESS Macro moderated mediation analyses with mean-centered participant political ideology (PPI) as a moderator in Study 4.

Effect	Outcome Variable	Mediator	1 SD below mean PPI (SE), [95% CI]	Mean PPI (SE), [95% CI]	1 SD above mean PPI (SE), [95% CI]	Index of moderated mediation (SE), [95% CI]
Direct	Respect	Rationality	B=.004(.14) [-.27, .28]	B=-.08(.11) [-.29, .14]	B=-.12(.14) [-.39, .16]	.03(.09) [-.15,.21]
Indirect	Respect	Rationality	B=.46(.19) [.08, .82]	B=.51(.16) [.21, .82]	B=.54(.21) [.13, .94]	
Direct	Interact	Rationality (M1) Respect(M2)	B=-.36(.18) [-.71, -.001]	B=-.22(.14) [-.50, .06]	B=-.15(.14) [-.51, .20]	.009(.03) [-.06,.07]
Indirect	Interact	Rationality (M1) Respect(M2)	B=.16(.09) [.02, .36]	B=.18(.08) [.05, .34]	B=.19(.09) [.03, .38]	

Note: Higher ratings on PPI = greater conservatism Models with 1 mediator were run using model 8, models with 2 mediators were run using model 85.
Based on Table S16, we see the mediation is not dependent on participant ideology. The indirect effect of the mediation model is significant regardless of participant ideology.

Interaction between participant ideology and condition: We also conducted analyses exploring whether participants stances on political topics (e.g., whether they are pro-gun or anti-gun) interacted with their ratings of rationality and respect. This was necessary to ensure there were no systematic differences in ratings based on participants' political stances. For each interaction term, we created a dichotomous variable based on the 6-point Likert measure participants used to report their stance on the policies. For each political topic (i.e., tax, coal, and gun policy), those participants who disagreed with the prompts were grouped together and were represented as 0 in the new dichotomous measures. Those who agreed with the prompt were also grouped together and represented as 1 in the new dichotomous measures This exploratory analysis was conducted for all subsequent studies where participants were asked their stance on a political topic. None of these analyses were pre-registered.

There was a non-significant interaction between participants' stances on tax policy and their responses to both the rationality, $F(1, 173)=2.55$, $p=.11$, and respect items $F(1,173)=.56$, $p=.46$. There was also a non-significant interaction between participants' stances on coal policy and their ratings of the target as rational, $F(1,173)=1.18$, $p=.28$, and the extent to which they respect the target, $F(1,173)=1.98$, $p=.16$. Further, there was not a significant interaction between participants' stances on gun policy and their views of the opponents as rational, $F(1,173)=.10$,

p=.76, and their willingness to respect them, $F(1,173)$=.22, p=.64. These non-significant interaction terms suggest participants' viewpoints on these topics did not impact how our conditions shaped their response to our measures.

Multilevel Analyses[10]. Since responses were nested within participants and further grouped by vignette type (the levels of which, tax, coal, and gun, were fully crossed with participants), we also estimated the effect of experience (vs. facts) via a multi-level model including random effects for both story type and participant id. We first ran null models entering only the random effects of participant id and story type predicting perceived rationality and respect. Intra-class correlations for participant id (ICC = .54, .56, and .64) for models predicting perceived rationality, respect, and willingness to interact, respectfully) and story type (ICC = .001, .007, and .015) suggested story type accounted for almost zero variance in the outcomes, thus all subsequent models include only the random effect of participant id (following recommendations from past research; *5*).

We next explored the effect of condition (i.e., facts vs. experience) on ratings of rationality and respect while allowing the intercepts for participant to vary. Analyses revealed that the fixed effects of experience on ratings of rationality, γ = .65, SE = .19, 95% CI [.27, 1.02], $t(174.89)$ = 3.39, p< .001, and respect, γ = .44, SE = .18, 95% CI [.10, 79], $t(175.14)$ = 2.53, p=.01, were both significant. However, the effect upon willingness to interact was nonsignificant, γ =-.03, SE=-.15, 95% CI [-.33, .26], $t(175)$ = -.202, p=.84. For perceived rationality, the model including the fixed effect of experience (AIC = 1835.2) had significantly better fit than the null model (AIC = 1844.4), $\chi^2(1)$=11.27, p<.001. When predicting respect, including experience also significantly improved the model fit (AIC = 1706.3) compared to the null model (AIC = 1710.6), $\chi^2(1)$=6.35, p=012. When predicting willingness to interact, the fit between the null (AIC = 1443.2) and fixed effects model (AIC = 1445.2) did not differ, $\chi^2(1)$=.04, p=.84

We next fit a 2-1-1 multilevel mediation model testing whether the between group effect of experience (vs. facts) (a level 2 variable) upon respect (level 1) was mediated by rationality (level 1). We followed Preacher and collogues recommendations (*6*) for separately examining between and within cluster effects in a structural equation modeling framework (fit with the Lavaan Survey package in R; *7*). Response level variables were group mean centered. Confidence intervals for indirect effects were calculated via Monte Carlo simulations with 10,000 iterations (*8*). The model had good fit, CFI=1, RMSEA=.00 ,90% CI [00.00]; SRMR=.00, 90% CI [.00, .00]; $\chi^2(1)$=.00, p=1.00. As predicted the between groups effect indirect effect of experience (vs evidence) upon respect, through rationality, was significant, indirect effect=.48, SE=.14, 95% CI [.21, .76], as was the total effect, total effect=.44, SE =.17, 95% CI [.10, .79]. The direct effect was not significant, direct effect=-.04, SE=.10, 95% CI [-24, .16].

We next fit a 2-1-1-1 serial mediation multilevel model to test the downstream effects upon willingness to interact. The model had good fit, CFI=1, RMSEA=.00 ,90% CI [00.00]; SRMR=.00, 90% CI [.00, .00]; $\chi^2(3)$=.002, p=1.00. The between groups, serial indirect effect of

[10] These multilevel analyses were not pre-registered.

experience (vs. facts) upon willingness to interact, through rationality and respect, was significant, indirect effect=.17, SE=.07, 95% CI [.13, .47], but the total effect was not, total effect=.14, SE=.20, 95% CI [-26, .54]. The effect of experience upon willingness to interact while controlling for respect and rationality (i.e., the direct effect) was negative and marginally significant, direct effect=-.24, SE=.13, *p*=.09, 95% CI [-51, .04]. Full results of the serial mediation model, including estimates of within cluster effects, are presented in Table S17.

Table S17

Full results of serial mediation model in Study 4

	Estimate(SE)	*p*-value/95%CI
Outcome: Willingness to Interact (Response Level)		
Experience	-0.24(0.14)	0.09[-.51,.05]
Rational Between	0.09(0.09)	0.33[-.09,.27]
Rational Within	0.05(0.06)	0.33[-.06,.16]
Respect Between	0.35(0.11)	0.002[.13,.56]
Respect Within	0.36(0.09)	<.001[.19,.53]]
Outcome: Rationality (Cluster Mean)		
Experience	0.65(0.19)**	.001[.27,1.02]
Outcome: Respect (Cluster Mean)		
Experience	-0.04(0.10)	0.66[-.24,.16]
Rational Between	0.74(0.04)**	<.001[.66,.83]
Rational Within	-0.00(0.00)	0.88[.00,.00]
Indirect Effect: Through Rationality	0.22(0.10)**	[.06,.44]
Indirect Effect: Through Respect	-0.02(0.04)	[-.10,.06]
Indirect Effect: Serial	0.17(0.07)**	[.04,.33]
Total Effect	0.14(0.20)	[-.26,.54]

Note: Since experience was manipulated at level 2, indirect effects are calculated by multiplying the between cluster effects. Confidence intervals for indirect effects are estimated via the Monte Carlo method.

Simple effects for each vignette. Lastly, we also explored the simple effects of condition within each story type, while still allowing the intercept for participant vary[11]. When predicting rationality, a model including the interaction between condition and story type (AIC = 18.36.5) did not improve fit relative to a model that including only main effects of condition and story types (AIC = 1836.2), $\chi^2(1)=6.35$, *p*=012. However, further analyses indicated a marginal interaction between condition and the coal vs. tax story contrast (estimate=.42, SE=.23, *p*=06), but no interaction between condition and the gun vs. tax story contrast (estimate=.28, SE=.23, *p*=.22). In order to more fully understand how story type interacted with condition (i.e., experience vs. facts), we also conducted simple slopes analysis. Analyses revealed there were significant differences in ratings of rationality between conditions in both the coal policy vignettes (estimate=.84, SE=.23, *p*<.001) and gun policy vignettes (estimate=.70, SE=.23, *p*<.001). There were trending differences with the tax policy vignettes (estimate=.42, SE=.23, *p*=.07). Conducting this same analysis with respect as the outcome, including the interaction between condition and story type also did not improve model fit (AIC = 1705.2) relative to a model containing only the main effects of condition and story type (AIC = 1702.9), $\chi^2(2)=1.47$, *p*=.48. Personal experience (vs. facts) lead to more respect for the gun story vignette (estimate=.42, SE=.21, *p*=.05), the coal vignette (estimate=.57, SE=.21, *p*=.01), and trended for the tax vignette (estimate=.34, SE=.21, *p*=.11).

[11] These analyses were not pre-registered.

Study 5: Field Study

Two sets of analyses were conducted, one with the confederates' codes of how they perceived the participant viewed them, and a second with blind to hypothesis research assistants who coded based on the recorded conversations between the participant and confederate. All analyses in the main text were in reference to the blind to hypothesis research assistants' codes. Below are analyses based on the confederates' codes, and additional analyses associated with the research assistants' codes.

Confederate Codes:
Table S18

Means for dependent measures of confederate ratings in Study 5

	Facts Means (SD)	Personal Experience Means (SD)	Inferential	Cohen's d
Rational	4.34(1.69)	5.21(1.56)	*t*(151)=-3.29, *p*<.001	0.53
Respect	5.20(1.50)	5.89(1.52)	*t*(151)=2.83, *p*=.005	0.46
Willingness to Interact	5.23(1.57)	5.85(1.46)	*t*(151)=-2.65, *p*=.009	0.41

Table S19

PROCESS Macro mediational analyses of confederate ratings in Study 5.

Outcome Variable	Mediator(s)	Indirect effect of Mediator(s) (SE), [95% CI]	Effect of Condition on Mediator (SE), [95% CI]	Effect of M1 on M2 (SE), [95% CI]	Effect of Mediator on DV (SE), [95% CI]	Total Effect (SE), [95% CI]	Direct Effect (SE), [95% CI]
Respect	Rationality	B=.63(.19), [.26, 1.00]	B=.87(.26), [.35, 1.39]		B=.72(.05), [.63, .81]	B=.69(.24), [.21, 1.18]	B=.06(.16), [-.25, .38]
Interact	Rationality (M1) Respect (M2)	B=.48(.16), [.19, .80]	B=.87(.26), [.35, 1.39]	B=.72(.05), [.63, .81]	B=.61(.23), [.16, 1.07]	B=.61(.23), [.16, 1.07]	B=.24(.18), [-.12, .60]

Effect of Location: To ensure the validity of results, a One-way ANOVA was conducted to ensure there were no significant differences between locations of participant recruitment. On all measures (i.e., rationality, respect, and interaction), there were no significant differences ($F(3,149)=.89$, $p=.45$, $F(3,149)=1.08$, $p=.36$, $F(3,149)=.27$, $p=.95$, respectively).

Reliability: Two sets of research assistants ran this field study (2 dyads with one confederate and one researcher in each). There were some differences in codes based on confederate. There were trending (but not significant) differences in ratings of rationality ($M1=5.00$, $SD1=1.74$, $M2=4.50$, $SD=1.60$) $t(151)=-1.85$, $p=.07$, and willingness to interact ($M1=5.73$, $SD1=1.45$, $M2=5.31$, $SD=1.44$), $t(151)=-1.82$, $p=.07$. However, these differences are not a concern as the blind to hypothesis research assistants' codes led to non-significant differences between these dyads (see next section for more information).

Interaction between participant ideology and condition. There was a significant interaction term between participant stance on gun policy and perceptions of rationality (as rated by confederates), $F(1,149)=6.17$, $p=.01$. There was a non-significant interaction between participant stance on guns and the condition, $F(1, 149)=2.46$, $p=.12$. However, only 4 individuals reported a pro-gun stance, and only one of those participants was in the factual knowledge condition—making these interaction terms less meaningful.

Blind to Hypothesis Research Assistant Codes:

Table S20

Means for dependent measures of blind to hypothesis codes in Study 5

	Facts Means (SD)	Personal Experience Means (SD)	Inferential	Cohen's d
Rational	3.30(1.04)	3.99(1.25)	$t(151)=-3.71$, $p<.001$	0.60
Respect	3.95(1.15)	4.55(1.12)	$t(151)=-3.71$, $p=.001$	0.53
Willingness to Interact	4.38(1.05)	4.95(.88)	$t(151)=-3.57$, $p<.001$	0.59

Table S21

PROCESS Macro mediational analyses of blind to hypothesis codes in Study 5.

Outcome Variable	Mediator(s)	Indirect effect of Mediator(s) (SE), [95% CI]	Effect of Condition on Mediator (SE), [95% CI]	Effect of M1 on M2 (SE), [95% CI]	Effect of Mediator on DV (SE), [95% CI]	Total Effect (SE), [95% CI]	Direct Effect (SE), [95% CI]
Respect	Rationality	B=.56(.16), [.26, .90]	B=.69(.19), [.32, 1.05]		B=.82(.05), [.73, .91]	B=.60(.18), [.24, .96]	B=.03(.11), [-.18, .25]
Interact	Rationality (M1) Respect (M2)	B=.41(.13), [.18, .70]	B=.69(.19), [.32, 1.05]	B=.82(.05), [.73, .91]	B=.57(.16), [.25, .88]	B=.57(.16), [.25, .88]	B=.23(.12), [-.004, .46]

Effect of Location*:* Surprisingly, there were significant differences by location site for ratings by coders for respect such that they rated respect much higher at one of the location sites (UNC Union, M=5.33), than at the other four locations (mean respect ~4.13 for all four locations), $F(149)=2.96$, p=.03. This is surprising as the coders were not aware of the test site location when completing coding. However, only 9 participants were collected at the UNC union, suggesting this is likely not systemic issue within the participant pool. For both rationality and willingness to interact, there were not significant differences based on location. Coders ratings were not significantly different from the confederates' ratings on rationality, $t(151)=.65$, p=.52, respect $t(151)=-.05$, p=.96, and willingness to interact $t(151)=-.63$, p=.53.

Reliability*:* The coders ratings were significantly correlated to one another for rationality $r=.62$, $p<.001$, respect $r=.62$, $p<.001$, and willingness to interact $r=.19$, $p=.02$.

Interaction between participant ideology and condition: We also tested the significance of the interaction terms between participant stance on guns, and blind to hypothesis research assistants' ratings of their perceived rationality and respect towards the confederate. There was a non-significant interaction between participant gun stances and condition on ratings of rationality (as rated by the blind to hypothesis research assistants), $F(1,149)=1.18$, $p=.28$. There was also not a significant interaction between participant stances toward gun policy and ratings of respect, $F(1, 149)=.57$, $p=.45$. However, again only 4 participants reported being pro-gun, and only one of those participants was in the factual knowledge condition—making meaningful interpretation of these interaction terms less meaningful.

Study 6: Specific Facts- Pilot Study

Table S22 indicates the factual items we retrieved from: https://www.justfacts.com/guncontrol.asp were viewed as highly specific—suggesting these prompts would be appropriate for testing the extent to which specificity could impact rationality and respect of adversaries.

Table S22

Means of perceived specificity for items tested in the Study 6 pilot study.

Prompts	Mean Perceived Specificity (SD)
Someone reads in an annual report that 73% of. Murders in the United States are committed with firearms†	5.12(1.05)
Someone shoots an intruder to protect his young daughter**	4.52(1.03)
Someone's young daughter is hit by a stray bullet**	4.38(1.03)
Someone reads in an annual federal report that civilians use guns to defend themselves over 989,000 times per year†	4.88(1.23)
Someone completes their annual tax return early	4.32(.99)
Someone leaps from a moving car	4.05(1.17)
Someone finds something	2.38(1.17)
Someone watches the Jeopardy gameshow at 7:30pm every Tuesday	5.08(1.22)
Someone calculates gas mileage using a Casio calculator	4.83(1.00)
Someone moves	2.53(1.12)

Note: †= Specific Factual items, **= Personal Experience items

Study 6: Specific Facts

Table S23 shows the experiential vignettes did indeed reflect more personal experience, and the factual vignettes reflected more factual knowledge. Further, the experiential vignettes elicited greater perceived rationality, respect, and willingness to interact.

Table S23

Mean Scores for manipulation check measures and dependent measures in Study 6.

	Facts Means (SD)	Personal Experience Means (SD)	Inferential	Cohen's d
Personal Experience	3.51(1.30)	5.87 (.98)	*t*(192) = 21.11, p < .001	2.05
Facts	4.26 (1.07)	3.68 (1.44)	*t*(192) = 10.73, p =.002	0.46
Rational	3.95(1.40)	4.90(1.36)	*t*(192) = 6.68, p <.001	0.69
Respect	4.45 (1.26)	5.34(1.13)	*t*(192) = 6.90, p < .001	0.74
Willingness to Interact	5.19 (1.38)	5.64 (1.21)	*t*(192) = 3.62, p =.018	0.34

Table S24

PROCESS Macro mediational analyses in Study 6.

Outcome Variable	Mediator(s)	Indirect effect of Mediator(s) (SE), [95% CI]	Effect of Condition on Mediator (SE), [95% CI]	Effect of M1 on M2 (SE), [95% CI]	Effect of Mediator on DV (SE), [95% CI]	Total Effect (SE), [95% CI]	Direct Effect (SE), [95% CI]
Respect	Rationality	B=.58(.13), [.33, .84]	B=.95(.20), [.56, 1.34]		B=.61(.04), [.52, .70]	B=.88(.17), [.54, 1.22]	B=.30(.13), [.05, .56]
Interact	Rationality (M1) Respect (M2)	B=.35(.11), [.17, .59]	B=.95(.20), [.56, 1.34]	B=.61(.04), [.52, .70]	B=.60(.10), [.41, .79]	B=.44(.19), [.08, .81]	B=-.01(.17), [-.35, .33]

Interaction between participant ideology and condition: There was a significant interaction between participants stances on gun policy and condition on ratings of rationality, $F(1, 190)=3.80$, $p=.05$. In general, participants who were pro-gun ($M=3.63$, $SD=1.35$), rated opponents as less rational than participants who were anti-gun ($M=4.67$, $SD=1.41$). There was a non-significant interaction between participant stance on guns and condition on willingness to respect opponents, $F(1, 190)=.40$, $p=.53$.

Study 7: Non-relevant Experiences

Table S25

Mean Scores for manipulation check measures and dependent variables of Study 7.

	Facts Means(SD)	Personal Experience Means(SD)	Non-Relevant Experience Means(SD)	Inferential
Personal Experience	4.33(1.46)[a]	5.74(1.18)[b]	3.82(1.31)[c]	$F(2,270) = 51.56$, p < .001
Facts	4.09(1.38)[a]	3.24(1.43)[b]	3.39(1.24)[b]	$F(2,270) = 10.29$, p < .001
Rational	3.74(1.40)[a]	4.86(1.60)[b]	3.60(1.42)[a]	$F(2,270) = 19.93$, p < .001
Respect	4.40(1.34) [a]	5.19(1.26)[b]	4.29(1.41)[a]	$F(2,270) = 10.90$, p < .001
Willingness to Interact	5.07(1.58) [a]	5.68(1.23)[b]	4.88(1.52)[a]	$F(2,270) = 7.49$, p = .001

Note: means without a superscript letter in common are significantly different from one another at $p < .05$.

PROCESS macro mediational analysis: Dummy codes (D1 and D2) were made since the predictor variable must be dichotomous for comparisons. One dummy code at a time was entered as a predictor into the mediation, while we covaried for the other—this made it possible to make comparisons between only two groups at a time[12].

Table S26

PROCESS Macro mediational analyses in Study 7.

Outcome Variable	Mediator(s)	Indirect effect of Mediator(s) (SE), [95% CI]	Effect of Condition on Mediator (SE), [95% CI]	Effect of M1 on M2 (SE), [95% CI]	Effect of Mediator on DV (SE), [95% CI]	Total Effect (SE), [95% CI]	Direct Effect (SE), [95% CI]
	**Comparing Relevant Experiences to Facts**						
Respect	Rationality	B=.68(.15) [.40, .98]	B=1.12(.22) [.69, 1.54]		B=.61(.04) [.53, .69]	B=.80(.20) [.41, 1.18]	B=.12(.15) [-.19, .42]
Interact	Rationality (M1) Respect (M2)	B=.46(.12) [.26, .71]	B=1.12(.22) [.69, 1.54]	B=.61(.04) [.53, .69]	B=.68(.07) [.53, .82]	B=.60(.21) [.18, 1.03]	B=.18(.19) [-.19, .55]
	**Comparing Relevant Experiences to Non-Relevant Experiences**						
Respect	Rationality	B=.77(.15) [.83, 1.69]	B=1.26(.22) [.83, 1.69]		B=.61(.04) [.53, .69]	B=.80(.20) [.41, 1.19]	B=.03(.16) [-.27, .34]
Interact	Rationality (M1) Respect (M2)	B=.52(.12) [.31, .78]	B=1.26(.22) [.83, 1.69]	B=.61(.04) [.53, .69]	B=.68(.07) [.53, .82]	B=.80(.22) [.37, 1.22]	B=.38(.19) [.01, .75]
	**Comparing Facts to Non-Relevant Experiences**						
Respect	Rationality	B=.09(.12) [-.17, .35]	B=.14(.22) [-.29, .58]		B=.61(.04) [.53, .69]	B=.01(.20) [-.39, .40]	B=.08(.15) [-.37, .21]
Interact	Rationality (M1) Respect (M2)	B=.06(.09) [-.11, .24]	B=.14(.22) [-.29, .58]	B=.61(.04) [.53, .69]	B=.68(.07) [.53, .82]	B=.19(.22) [-.23, .62]	B=.20(.18) [-.15, .56]

Note: First two sets of mediations, facts and the non-relevant experiences are the reference group. In third set of mediations, facts are the reference group.

[12] This dummy coding strategy was also used in Studies, 8, 9, & 14.

In general, the mediation analyses suggest relevant personal experiences are significant better at driving rationality, respect, and subsequent willingness to interact than both facts and relevant experiences. The null results when non-relevant experiences and factual knowledge suggests they are viewed as one in the same—and that relevant experiences are uniquely effective at increasing rationality, respect, and willingness to interact with political opponents.

Interaction between participant ideology and condition: There was a non-significant interaction between participant stance on gun policy and condition on rations of rationality towards the opponent, $F(2, 267)=1.86$, $p=.16$. There was also a non-significant interaction between whether a participant was pro-gun or anti-gun and condition on respect towards opponents, $F(2, 267)=.41$, $p=.67$. Taken together, this suggests the effect of the condition did not differentially impact ratings of rationality and respect based on participant stances on gun policy.

Study 8: Non-Harmful Experiences

Table S27

Mean Scores for manipulation check measures and dependent variables of Study 8.

	Facts Means(SD)	Personal Experience Means(SD)	Non-Relevant Experience Means(SD)	Inferential
Personal Experience	2.49(1.51)[a]	5.89(1.07)[b]	4.94(1.31)[c]	$F(2,252) = 151.96$, p < .001
Facts	4.12(1.41)[a]	3.11(1.67)[b]	3.56(1.30)[c]	$F(2,252) = 9.98$, p < .001
Rational	3.44(1.62)[a]	5.00(1.42)[b]	4.09(1.57)[c]	$F(2,252) = 22.21$, p < .001
Respect	3.95(1.65) [a]	5.34(1.21)[b]	4.55(1.41)[c]	$F(2,252) = 20.09$, p < .001
Willingness to Interact	4.69(1.77) [a]	5.69(1.12)[b]	5.40(1.24)[a]	$F(2,252) = 12.04$, p < .001

Note: means without a superscript letter in common are significantly different from one another at $p < .05$.

Table S28

PROCESS Macro mediational analyses in Study 8.

Outcome Variable	Mediator(s)	Indirect effect of Mediator(s) (SE), [95% CI]	Effect of Condition on Mediator (SE), [95% CI]	Effect of M1 on M2 (SE), [95% CI]	Effect of Mediator on DV (SE), [95% CI]	Total Effect (SE), [95% CI]	Direct Effect (SE), [95% CI]
		**Comparing Harmful Experiences to Facts**					
Respect	Rationality	B=1.01(.17) [.69, 1.37]	B=1.56(.24) [1.10, 2.02]		B=.65(.04) [.56, .73]	B=1.38(.22) [.95, 1.81]	B=.37(.17) [.04, .71]
Interact	Rationality(M1) Respect(M2)	B=.63(.13) [.39, .91]	B=1.56(.24) [1.10, 2.02]	B=.65(.04) [.56, .73]	B=.62(.07) [.48, .76]	B=1.00(.21), [.58, 1.42]	B=.30(.19), [-.08, .68]
		**Comparing Harmful Experiences to Non-Harmful Experiences**					
Respect	Rationality	B=.59(.15) [.29, .90]	B=.91(.23) [.45, 1.36]		B=.64(.04) [.56, .73]	B=.79(.22) [.36, 1.22]	B=.20(.16) [-.11, .52]
Interact	Rationality(M1) Respect(M2)	B=.37(.10) [.18, .58]	B=.91(.23) [.45, 1.36]	B=.64(.04) [.56, .73]	B=.68(.07) [.48, .76]	B=.20(.21) [-.22, .61]	B=-.20(.18) [-.56, .15]
		**Comparing Facts to Non-Harmful Experiences**					
Respect	Rationality	B=-.42(.16) [-.74, -.10]	B=-.65(.24) [-1.12, -.18]		B=.64(.04) [.56, .73]	B=-.59(.22) [-1.03, -.15]	B=-.17(.16) [-.49, .15]
Interact	Rationality(M1) Respect(M2)	B=-.26(.11) [-.49, -.07]	B=-.65(.24) [-1.12, -.18]	B=.64(.04) [.56, .73]	B=.62(.07) [.48, .76]	B=-.80(.22) [-1.23, -.38]	B=-.50(.18) [-.86, -.15]

Note: First two sets of mediations, facts and the non-harm experiences are the reference group. In third set of mediations, non-harm experiences are the reference group.

Interaction between participant ideology and condition: There was a non-significant interaction between participant stances on gun policy and condition on ratings of rationality, $F(2, 249)=1.47$, $p=.23$. There was also not a significant interaction between gun policy stances and condition on willingness to respect the target opponent, $F(2, 149)=.66$, $p=.52$. These results suggest the effect of condition on ratings of rationality and respect were similar across viewpoints on gun policy.

Study 9: Personal Experience: Pilot Study
The higher the rating, the more similar to the self it was perceived. There was a main effect of personal experience across ratings of items, $F(4, 188)=38.99$, $p<.001$, see Table S29. This main effect suggested overall significant differences in ratings of the specific relationships. The self was rated higher (M=5.90, SD=1.75), than a brother (M=4.08, SD=1.74), a friend (M=4.06, SD=1.68), somebody they read about in a book (M=2.79, SD=1.82), and an acquaintance living in another state (M=2.71, SD=1.75) in terms of similar.

Table S29

Means of perceived similarity to the self for items tested in the Study 9 pilot study.

Test Items	Mean Perceived Similarity to Self (SD)
The Self	5.90(1.75)
A Brother	4.08(1.74)
A Friend	4.06(1.68)
An acquaintance living in another state	2.71(1.75)
Somebody they read about in a book	2.79(1.82)

Study 9: Personal Experience

Table S30

Means and main effects for measures across personal experience gradient items in Study 9.

	Self (SD)	Brother (SD)	Friend (SD)	Acquaintance (SD)	Book Character (SD)	Inferential
Personal Experience	5.74(.92) [a]	5.32(1.11) [b]	4.98(1.41) [b]	4.53(1.47) [c]	2.77(1.72) [d]	$F(4,403) = 60.33$, $p < .001$
Facts	3.35(1.46) [a]	3.00(1.56) [a]	3.08(1.46) [a]	3.23(1.63) [a]	3.33(1.37) [a]	$F(4,403) = 0.90$, $p = .46$
Rational	4.68(1.31) [a]	4.25(1.37) [ab]	4.37(1.62) [ab]	4.20(1.45) [b]	3.53(1.55) [c]	$F(4,403) = 6.92$, $p < .001$
Respect	5.08(1.29) [a]	4.75(1.20) [ab]	4.66(1.36) [b]	4.70(1.28) [ab]	4.21(1.29) [c]	$F(4,403) = 4.79$, $p = .001$
Interact	5.39(1.17) [ab]	5.62(1.18) [a]	5.56(1.05) [ab]	5.19(1.46) [b]	5.53(1.23) [ab]	$F(4,403) = 1.59$, $p = .18$

Note: means without a superscript letter in common are significantly different from one another at $p < .05$.

PROCESS macro mediational analyses:
A mediation analyses was conducted comparing ratings of the highly experiential target (i.e., the self), and the target low in experience (i.e., somebody the target read about in a book). The three other targets varying in experience were added as covariates in the model, and the highly experiential target was the reference group.

Table S31

PROCESS Macro mediational analyses in Study 9.

Outcome Variable	Mediator(s)	Indirect effect of Mediator(s) (SE), [95% CI]	Effect of Condition on Mediator (SE), [95% CI]	Effect of M1 on M2 (SE), [95% CI]	Effect of Mediator on DV (SE), [95% CI]	Total Effect (SE), [95% CI]	Direct Effect (SE), [95% CI]
Respect	Rationality	B=-.67(.13) [-.95, -.42]	B=-1.15(.23) [-1.60, -.70]		B=.59(.03) [.52, .65]	B=-.86(.20) [-1.26, -.47]	B=-.19(.15) [-.49, .11]
Interact	Rationality (M1) Respect(M2)	B=-.34(.08) [-.51, -.20]	B=-1.15(.23) [-1.60, -.70]	B=.59(.03) [.52, .65]	B=.50(.06) [.39, .61]	B=.13(.19), [-.24, .51]	B=.49(.17), [.15, .83]

Note: The highly experiential target was the reference group.

Interaction between participant ideology and condition: There was a non-significant interaction between participant stance on gun policy and condition on ratings of rationality, $F(4, 398)=.41$, $p=.80$. Further, there was not a significant interaction between whether participants had a pro- or anti-gun stance on ratings of respect, $F(4, 398)=.24$, $p=.92$. These results suggest the effect of condition on ratings of rationality and respect did not differ based on whether the participant was pro-gun or anti-gun.

Study 10: Real Experiences vs. Real Facts- Step 2 (Controlling for Harm)
There was a significant moderate correlation between the compelling and harm measures, $r(220)=.49$, $p<.001$. The means from the harm measure were used to determine which pro- and anti-gun experiences were viewed as similarly harmful. We paired 3 sets of low, medium, and high harm experiences together (See Table S32) which were used as the real experiences in the final part of this study (See next section).

Table S32

Mean Scores for Study 10 Step 2 (Real Facts Harm Ratings).

Pro-gun Participant Experiences	Harm Ratings Mean (SD)	Anti-gun Participant Experience	Harm Ratings Mean (SD)
**Low Harm:** Guns do not kill people, its the people control the gun that kills people. We should have tighter check on people who uses the weapon and not limiting the weapon.	3.49(1.46)	**Low Harm:** I believe that limiting access to guns will save lives and reduce crimes in the us. I experience a neighbor who has guns he got really upset about something and then he fired his gun randomly up in the sky. Nobody got hurt but there is a possibility that he would do it again and he might hurt somebody.	3.58(1.40)
**Medium Harm:** Every American without a criminal record should be able to own a gun. My Aunt was robbed, and she owned a gun. She was not home at the time, but the officer told her to shoot the their. If she was by herself and did not have the right to own a gun she could have been killed.	4.70(1.27)	**Medium Harm:** As a student, I had practiced many times for what to do if there were an active shooter on campus. I should be able to go to school without this worry. I also went to see the Batman film on the same night that someone had shot up the theater at another location. I should not have to fear going out in public.	4.68(1.03)
**High Harm:** One of my family friends was murdered in a home invasion robbery. If she was legally allowed to have a gun to protect herself it may have been prevented. Guns are part of the Constitution.	5.80(0.90)	**High Harm:** I had a friend who was shot and murdered by a gun. The person who killed him was able to get that gun without having a proper background check. Gun laws need to be change.	5.80(1.20)

Note: Since these were the actual experiences written by MTurkers in Study 10 Step 1, there were typos in many of the vignettes. Participants in this section of the study were warned of the possibility of typos at the beginning of the study.

Study 10: Real Experiences vs. Real Facts- Step 3 (Comparing Experiences and Facts)

Table S33

Mean Scores for manipulation check measures and dependent variables of Study 10.

	Facts Means (SD)	Low Experience Means(SD)	Medium Experience Means(SD)	High Experience Means(SD)	Inferential
Personal Experience	3.07(1.46)[a]	4.45(1.36)[b]	4.48(1.51)[b]	5.45(1.10)[c]	$F(3,1560)=224.97$, $p<.001$
Facts	4.16(1.31)[a]	3.22(1.56)[b]	2.98(1.51)[b]	3.04(1.51)[b]	$F(3,1558)=75.57$, $p<.001$
Doubt	3.98(1.44)[a]	4.18(1.44)[b]	4.37(1.40)[b]	4.79(1.30)[c]	$F(3,1560)=23.47$, $p<.001$
Rational	3.79(1.58)[a]	3.98(1.40)[ab]	4.06(1.59)[b]	4.35(1.58)[c]	$F(3,1556)=8.45$, $p<.001$
Respect	4.28(1.45)[a]	4.55(1.53)[b]	4.62(1.36)[b]	4.98(1.35)[c]	$F(3,1559)=16.41$, $p<.001$
Willingness to Interact	5.16(1.49)[a]	5.46(1.43)[b]	5.32(1.54)[a]	5.52(1.35)[b]	$F(3,1560)=5.49$, $p=.001$

Note: means without a superscript letter in common are significantly different from one another at $p < .05$. Higher scores on doubt measure indicate less doubt (i.e., greater perceived truth).

PROCESS macro mediational analysis:
In this series of mediations, we collapsed across all factual knowledge conditions (reference group), and collapsed across all personal experience conditions for comparison. Overall, doubt mediated the relationship between condition and rationality, rationality mediated the link between condition and respect, and the mediational pathway between rationality and respect mediated the link between condition and willingness to interact.

Table S34

PROCESS Macro mediational analyses in Study 10.

Outcome Variable	Mediator(s)	Indirect effect of Mediator(s) (SE), [95% CI]	Effect of Condition on Mediator (SE), [95% CI]	Effect of M1 on M2 (SE), [95% CI]	Effect of Mediator on DV (SE), [95% CI]	Total Effect (SE), [95% CI]	Direct Effect (SE), [95% CI]
Rationality	Doubt	B=.42(.07), [.29, .55]	B=.46(.07), [.32, .60]		B=.92(.02), [.88, .95]	B=.34(.08), [.18, .50]	B=.08(.05), [-.18, .01]
Respect	Rationality	B=.21(.05), [.11, .31]	B=.34(.08), [.18, .50]		B=.63(.02), [.60, .66]	B=.43(.07), [.29, .58]	B=.22(.05), [.12, .33]
Interact	Rationality (M1) Respect (M2)	B=.12(.03), [.06, .18]	B=.34(.08), [.18,.50]	B=.63(.02), [.60, .66]	B=.57(.03), [.51, .63]	B=.27(.07), [.13, .42]	B=.03(.06), [-.10, .15]

Interaction between participant ideology and condition: There was a significant interaction between stance on gun policy and condition on ratings of rationality, $F(1, 1036)=5.36$, $p=.02$. In general, pro-gun participants viewed opponents as less rational ($M=3.67$, $SD=1.67$), as compared to anti-gun participants ($M=4.03$, $SD=1.56$). There was a non-significant (but trending) interaction between participant gun stance and condition on ratings of respect, $F(1, 1037)=2.98$, $p=.09$. These results suggest participant gun stance led to differential responses across conditions.

Study 11: Real Op-ed Articles: Perceptions of Authors—Pilot Study

The pilot study indicated the articles we selected highlighting experience did indeed highlight experience and those we deemed to highlight facts were viewed as such by participants in the pilot study.

Table S35

Mean scores from Study 11 pilot study

	Fact Articles Means (SD)	Experience Articles Means (SD)	Inferential	Cohen's d
Personal Experience	3.63(1.63)	6.32(.93)	$t(65)$ =10.55, p <.001	2.02
Facts	5.92(.93)	3.38(1.83)	$t(65)$= -9.79, p < .001	1.75

Study 11: Real Op-ed Articles: Perceptions of Authors

Table S36

Mean Scores for Study 11.

	Facts Means (SD)	Personal Experience Means (SD)	Inferential	Cohen's d
Rational	4.13(1.84)	4.95(1.56)	$t(423)$ =-4.99, p < .001	0.48
Respect	4.61(1.73)	5.43(1.23)	$t(423)$= -5.60, p < .001	0.54
Willingness to Interact	5.15(1.68)	5.70(1.27)	$t(423)$= -3.78, p < .001	0.37

Note: In this study participants were not asked the manipulation check questions (i.e., personal experience and facts).

Table S37

PROCESS Macro mediational analyses in Study 11.

Outcome Variable	Mediator(s)	Indirect effect of Mediator(s) (SE), [95% CI]	Effect of Condition on Mediator (SE), [95% CI]	Effect of M1 on M2 (SE), [95% CI]	Effect of Mediator on DV (SE), [95% CI]	Total Effect (SE), [95% CI]	Direct Effect (SE), [95% CI]
Respect	Rationality	B=.54(.12), [.31, .78]	B=.82(.17), [.50, 1.15]		B=.66(.03), [.60, .72]	B=.81(.15), [.53, 1.10]	B=.27(.10), [.08, .47]
Interact	Rationality (M1) Respect (M2)	B=.31(.08), [.17, .47]	B=.82(.17), [.50, 1.15]	B=.66(.03), [.60, .72]	B=.57(.06), [.45, .68]	B=.54(.14), [.26, .83]	B=.04(.12), [-.19, .27]

Interaction between participant ideology and condition: There was a significant interaction term between participant stance on gun policy and condition on evaluations of rationality of the op-ed writer, $F(1, 421)=3.99$, $p=.05$. In general, pro-gun participants rated the writer as less rational ($M=3.74$, $SD=1.85$), as compared to anti-gun participants ($M=4.88$, $SD=1.60$). There was also a significant interaction term between stances on gun policy and condition on ratings of respect, $F(1, 421)=9.36$, $p=.002$. On average, pro-gun participants were less respectful ($M=4.61$, $SD=1.76$), as compared to anti-gun participants ($M=5.20$, $SD=1.43$).

Study 12: News Transcripts

Table S38

Correlations between coded variables

	M(SD)	1.	2.	3.	4.	5.	6.
1.Experience	3.06(1.83)	1.00					
2.Facts	4.10(1.92)	-.48**	1.00				
3.Rationality	4.69(1.59)	.14	-.20*	1.00			
4.Respect	5.70(1.35)	.10	-.01	.77**	1.00		
5.Interact	6.00(1.13)	.16	-.10	.75**	.78**	1.00	
6. Composite Score (Experience - Fact)	-1.04(3.23)	.85**	-.87**	.20*	.06	.15	1.00

Note: ** represents correlation at the p<.01 level, * represents correlation at the p<.05 level

Table S39

PROCESS Macro mediational analyses in Study 12.

Outcome Variable	Mediator(s)	Indirect effect of Mediator(s) (SE), [95% CI]	Effect of Condition on Mediator (SE), [95% CI]	Effect of M1 on M2 (SE), [95% CI]	Effect of Mediator on DV (SE), [95% CI]	Total Effect (SE), [95% CI]	Direct Effect (SE), [95% CI]
Respect	Rationality	B=.06(.03), [.01, .12]	B=.10(.04), [.01, .18]		B=.67(.05), [.58, .77]	B=.02(.04), [-.05, .09]	B=-.04(.02), [-.09, .004]
Interact	Rationality (M1) Respect (M2)	B=.03(.01), [.004, .05]	B=.10(.04), [.01, .18]	B=.67(.05), [.58, .77]	B=.42(.07), [.29, .56]	B=.05(.03), [-.01, .11]	B=.02(.02), [-.02, .05]

Table S40

Differences in mean scores of codes between Fox and CNN transcripts in Study 12.

	Fox Means (SD)	CNN Means (SD)	Inferential	Cohen's d
Personal Experience	3.04(1.77)	3.08(1.91)	*t*(135) =-.14, p=.89	.02
Facts	4.09(2.04)	4.11(1.81)	*t*(135)= -07, p=.94	.01
Rationality	4.45(1.53)	4.94(1.62)	*t*(135)=-1.83, p=.07	.31
Respect	5.36(1.43)	6.05(1.17)	*t*(135)=-3.08, p=.002	.53
Interact	5.88(1.01)	6.12(1.24)	*t*(135)=-1.25, p=.21	.21

Study 13: Doubt and Moral vs Non-Moral Contexts

Overall, personal experience was rated higher amongst the personal experience epistemological conditions, $F(7, 535)=116$, $p<.001$. Factual knowledge was rated higher in the factual epistemological conditions, $F(7, 535)=7.92$, $p<.001$. In general, people were less likely to doubt characters in experiential conditions, $F(7, 535)=27.34$, $p<.001$.People tended to view experiential characters as more rational, $F(7, 535)=21.71$, $p<.001$, were more willing to respect them $F(7, 535)=19.35$, $p<.001$, and were also more willing to interact, $F(7, 535)= 4.53$, $p<.001$

Interactions between participant ideology and condition. With a 2x2x2 study design, there are a multitude of interactions we could test. We decided the key interactions to explore would be participants political stances and whether the condition was based on facts or experience on rationality and respect—both within in moral (e.g., gun policy) and non-moral contexts (e.g., preferences for blenders).Within the moral domain, participants view on tax policy did not significantly interact with condition (i.e., whether the target based their stance on facts or experience), on ratings of rationality, $F(1, 262)=.70$, $p=.40$ or respect, $F(1, 262)=.09$, $p=.77$. Further, there was not a significant interaction between participant stances on coal policy and condition on ratings of rationality, $F(1, 262)=2.78$, $p=.10$, or respect, $F(1,262)=1.02$, $p=.31$. There also was not a significant interaction between participant views on guns and condition on ratings of rationality, $F(1, 262)=2.88$, $p=.09$, or respect, $F(1, 262)=.19$, $p=.67$.

Within the non-moral domain, participants preferences for financial decisions and condition (i.e., whether the target based their stance on facts or experience), did not significantly interact on ratings of rationality, $F(1, 273)=.16$, $p=.69$, or respect, $F(1, 273)=1.03$, $p=.31$. Furthermore, preferences for a diesel or gasoline car and condition did not produce a significant interaction both for rationality, $F(1, 273)=1.22$, $p=.27$, and respect, $F(1, 273)=.02$, $p=.89$. Finally, there was not a significant interaction between participants preferences for certain kinds of blenders and condition on ratings of rationality, $F(1, 273)=.01$, $p=.91$, and respect, $F(1, 273)=.50$, $p=.48$.

Taken together, these interactions suggest that regardless of whether opinions were about moral or non-moral topics, participants stances did not differentially impact ratings of rationality and respect across conditions.

Interactions between experience vs facts, moral vs non-moral, and target agreement vs disagreement on dependent variables. There was a non-significant interaction between conditions (i.e., whether the vignette was based in facts or experiences, moral or non-moral contexts, and whether the target agreed or disagreed) on the dependent variables. Specifically, on lower perceived truth (i.e., greater doubt) $F(1, 535)=3.12$, $p=.08$, rationality $F(1, 535)=1.14$, $p=.29$, and respect $F(1, 535)=2.67$, $p=.10$.

Table S41

PROCESS Macro mediational analyses in Study 13.

Outcome Variable	Mediator	Indirect effect of Mediator(s) (SE), [95% CI]	Effect of Condition on Mediator (SE), [95% CI]	Effect of Mediator on DV (SE), [95% CI]	Total Effect (SE), [95% CI]	Direct Effect (SE), [95% CI]
Rationality	Doubt	B=.81(.10), [.62, 1.00]	B=.80(.07), [.62, .99]	B=1.00(.02), [.96, 1.04]	B=.58(.11), [.39, .81]	B=-.21(.05), [-.31, -.12]
Rationality	Doubt	B=1.22(.15), [.92, 1.51]	B=1.20(.10), [.91, 1.49]	B=1.02(.03), [.96, 1.07]	B=.96(.17), [.64, 1.29]	B=-.26(.08), [-.41, -.10]

Note: The first model collapsed across all conditions (i.e., both moral and non-moral topics). The second model only focused on comparisons between conditions with moral topics. Higher scores on the doubt measure represent less doubt (i.e., greater perceived truth)

Multilevel Analyses. We also conducted multi-level model analyses with vignette type (tax, coal, and gun) and participant entered as random effects[13]. We conducted these analyses to supplement our pre-registered analyses plan with models that were better suited for handling clustering in our data structure and for understanding how results varied across story type. We first ran null effects models entering only the random effects of story type and participant id. Based on the variance components of this model, we calculated the interclass coefficient (ICC) in order to determine the necessity for conducting further multi-level analyses. Intra-class correlations for participant id (ICC = .58, .56, and .57 for models predicting perceived rationality and respect and doubt, respectfully) and story type (ICC = .01, .01, and .02) suggested story type accounted for nearly zero variance in the outcomes, thus all subsequent models include only the random effect of participant id (following recommendations from *38*).

[13] Multi-level analyses were not pre-registered.

We first explored the effect of condition (i.e., facts vs. experience) on ratings of rationality and respect while allowing the intercepts for participant to vary. Examining doubt (higher scores less doubt) the fixed effects of experience, γ = .86, SE = .09, 95% CI [.68,1.04], t(539.10) = 9.26, p< .001, and disagreement, γ = -.60, SE = .09, 95% CI [-.78, -.42] , t(539.10) = -6.43, p< .001, were both significant. However, the moral (vs. nonmoral) condition did not affect doubt, γ =.12, SE=.09, 95% CI [-.06, .30], t(539.04) = 1.30, p=.19. Examining rationality, the fixed effects of experience, γ = .66, SE = .10, 95% CI [.47, 87], t(538.63) = 6.52, p< .001, and disagreement, γ = -76., SE = .10, 95% CI [-.96,-.56] , t(538.61) = -7.43, p< .001, were both significant. However, the moral (vs. nonmoral) condition did not affect rationality, γ =.16, SE=.10, 95% CI [.10, .79], t(538.77)= 1.61, p=.11. Finally, when examining respect, the fixed effects of experience, γ = .47, SE = .09, 95% CI [.29, .64], t(539.29) = 5.25, p< .001, disagreement, γ = -.61, SE = .09, 95% CI [-.79, -.44] , t(539.36) = -6.43, p< .001, and the moral (vs. nonmoral) condition, γ =.24, SE=.09, 95% CI [.07, .41], t(539.44) = 1.30, p=.007, were significant.

Next, we repeated our mediation analyses using a 2-1-1 multilevel mediation model, with experience (vs. facts) manipulated at level 2, doubt assed at level 1 and rationality assessed at level 1. Doubt was cluster mean centered and entered in addition to the cluster (i.e., participant) means for doubt to tease apart between and within effects (following previous recommendations; *9*). However, since our manipulation was at level 2, indirect effects were calculated by multiplying the appropriate between effects of condition and doubt. Agreement and Moral (vs. nonmoral) were entered as control variables. Confidence intervals for indirect effects were calculated using Monte Carlo simulations. Models were fit in the Lavaan.Survey package in R. The model had good fit, CFI=1, RMSEA=.00 ,90% CI [00.00]; SRMR=.00, 90% CI [.00, .00]; $\chi^2(1)$=.001, p=.98. As predicted the between groups effect on the indirect effect of experience (vs facts) upon rationality, through doubt, was significant, indirect effect=.84, SE=.09, 95% CI [.66, .1.02], as was the total effect, total effect=.66, SE =.10, 95% CI [.46, .86]. The direct effect was significant and negative, direct effect=-.18, SE=.05, 95% CI [-.28, .09]. See Table S42 for full results.

Table S42

Full results of multilevel condition to doubt to rationality mediation model (including nonmoral conditions).

	Estimate(SE)	*p-value*/95% CI
Outcome: Rationality (Cluster mean centered)		
Experience	-.18(.05)	<.001[-.28, -.09]
Doubt Between	.98(.02)	<.001[.94,1.02]
Doubt Within	.80(.03)	<.001[.74,.87]
Agreement (1=agree, 2=disagree)	-.17(.05)	<.001[-.26, -.08]
Moral or Nonmoral (1=nonmoral, 2=moral)	.05(.05)	0.28[-.04,.14]
Outcome: Doubt (Cluster means)		
Experience	.86(.09)	<.001[.68,1.04]
Agreement (1=agree, 2=disagree)	-.60(.09)	<.001[-.79, -.42]
Moral or Nonmoral (1=nonmoral, 2=moral)	.13(.09)	0.18[-.06,.31]
Indirect and Total Effects		
Indirect Effect	.84(.09)	[.66,1.02]
Total Effect	.66(.10)	[0.46,.87]

Note: Since experience was manipulated at level 2, the indirect effect was calculated by multiplying the effect of experience by the between cluster effect of doubt on rationality. Confidence intervals for the indirect effect were estimated via the Monte Carlo method.

We then repeated the same mediation analysis looking specifically within response to moral issues. Again, the indirect effect of experience upon rationality, through doubt, was significant, indirect effect=1.19, SE=.13, 95% CI[.94,1.43], as was the total effect, total effect=1.00, SE=.14, 95% CI[.72,1.28]. The direct effect was significant and negative, direct effect=-.19, SE=.08, 95% CI[-.34,-.04].

We repeated a similar procedure but this time exploring whether rationality mediated the relationship between experience and respect. Again, experience (vs. facts) was manipulated at level 2, rationality assed at level 1 and respect assessed at level 1. Rationality was cluster mean centered and entered in addition to the cluster (i.e., participant) means for rationality to tease apart between and within effects. Indirect effects and confidence intervals were calculated in the same way as the previous mediations. Agreement and Moral (vs. nonmoral) were again entered as control variables. The model had good fit, CFI=1, RMSEA=.00 ,90% CI [00.00]; SRMR=.00, 90% CI [.00, .00]; $\chi^2(1)$=.000, p=.99. As predicted the between groups effect on the indirect effect of experience (vs facts) upon respect, through rationality, was significant, indirect effect=.45, SE=.07, 95% CI [.31, .60], as was the total effect, total effect=.46, SE =09, 95% CI [.28, .63]. The direct effect was not significant, direct effect=.01, SE=.06, 95% CI [-.11, .12]. See Table S43 for full results.

Table S43

Full results of multilevel condition to rationality to respect mediation model (including nonmoral conditions).

	Estimate(SE)	*p-value*/95% CI
Outcome: Rationality (Cluster mean centered)		
Experience	-.18(.05)	<.001[-.28, -.09]
Doubt Between	.98(.02)	<.001[.94,1.02]
Doubt Within	.80(.03)	<.001[.74,.87]
Agreement (1=agree, 2=disagree)	-.17(.05)	<.001[-.26, -.08]
Moral or Nonmoral (1=nonmoral, 2=moral)	.05(.05)	0.28[-.04,.14]
Outcome: Doubt (Cluster means)		
Experience	.86(.09)	<.001[.68,1.04]
Agreement (1=agree, 2=disagree)	-.60(.09)	<.001[-.79, -.42]
Moral or Nonmoral (1=nonmoral, 2=moral)	.13(.09)	0.18[-.06,.31]
Indirect and Total Effects		
Indirect Effect	.84(.09)	[.66,1.02]
Total Effect	.66(.10)	[0.46,.87]

Note: Since experience was manipulated at level 2, the indirect effect was calculated by multiplying the effect of experience by the between cluster effect of doubt on rationality. Confidence intervals for the indirect effect were estimated via the Monte Carlo method.

Simple effects by story type. We next estimated the simple effects of experience (vs. facts), agree (vs. disagree), and moral (vs. nonmoral) upon rationality within each story type while still allowing the intercept for participant to vary[14]. Including the interaction between story type and each level 2 fixed effect significantly improved the model (AIC = 5315.0, $\chi^2(8)=61.49$, $p<.001$) relative to a model only including the fixed effects with no interactions (AIC = 5360.5). Further, the fixed effects of facts vs. experience and whether the target agreed with the target, significantly shaped ratings of rationality. The interaction between experience and the tax vs. coal policy contrast was significant (B=.35, SE=.12, $p=.003$); however, analyses comparing ratings of rationality across stories in both the experience and fact conditions revealed there were significant effects of experience in all three vignettes, (tax policy vignettes; B=.56, SE=.12, $p<.001$, coal policy vignettes; B=.89, SE=.12, $p<.001$, and gun policy vignettes; B=.54, SE=.12, $p<.001$). Interactions between experience (vs. facts) and both story type dummy contrasts (gun vs. coal: B=-.35, SE=.12, $p=.003$; and gun vs. tax: B=-.543, SE=.12, $p<.001$) were significant. However, all the effect of agreement was significant within all three vignettes (tax policy vignettes; B=-.50, SE=.12, $p<.001$, coal policy vignettes; B=-.85, SE=.12, $p<.001$, and gun policy vignettes; B=-.93, SE=.12, $p<.001$). Finally, the simple slopes analyses indicated no significant differences across all 3 vignettes in ratings of rationality between morality conditions (i.e., whether the topic was moral or non-moral), tax policy vignettes; B=.18, SE=.12, $p=.14$,

---

[14] These analyses were not pre-registered.

coal policy vignettes; B=.12, SE=.12, *p*=.34, and gun policy vignettes; B=.20, SE=.12, *p*=.10). Thus, while effects did vary in size to some degree across vignettes, the effects within each vignette were consistently significant (or non-significant in the case moral vs. nonmoral) in the same direction across vignettes.

We then conducted identical analyses with doubt entered as the outcome. Including the interaction between story type and each level 2 fixed effect significantly improved the model (AIC = 5041.3, $\chi^2(6)45.90$, *p*<.001) relative to a model only including the fixed effects with no interactions (AIC = 5075.2.5). The interaction between experience and the tax vs. coal policy contrast was significant (B=.60, SE=.11, *p*<.001); however, analyses comparing ratings of rationality across stories in both the experience and fact conditions revealed there were significant effects of experience in all three vignettes, (tax policy vignettes; B=.75, SE=.11, *p*<.001, coal policy vignettes; B=1.15, SE=.11, *p*<.001, and gun policy vignettes; B=.69, SE=.11, *p*<.001). Interactions between agree (vs. disagree) and both story type dummy contrasts (gun vs. coal: B=-.42, SE=.11, *p*<.001; and gun vs. tax: B=-.44, SE=.11, *p*<.001) were significant. However, the effect of agreement was significant within all three vignettes (tax policy vignettes; B=-.75, SE=.11, *p*<.001, coal policy vignettes; B=-.73, SE=.11, *p*<.001, and gun policy vignettes; B=-.31, SE=.11, *p*=.01). Finally, moral (vs nonmoral) tone increased doubt in the tax policy vignettes; B=.25, SE=.11, *p*=.03, but not in the coal policy vignettes; B=-.01, SE=.13, *p*=.90, and gun policy vignettes; B=.13, SE=.11, *p*=.25). The effect of moral tone on rationality was significantly greater in the tax (vs. gun) vignette, B=-.26, SE=.11, *p*=.02, but the effect did not differ across the tax and coal vignettes, B=-.12, SE=.11, *p*=.27. Thus, while effects did vary in size to some degree across vignettes, the effects within each vignette were consistent overall.

Study 14: Replication with Black Female Opponents: Pre-Test

Table S44

Mean ratings of vignettes across each condition for anti-gun rights vignettes in pre-test

Vignette	Specificity	Evocative	Salient
Experience	5.29(1.05) [a]	4.93(1.45) [a]	5.90(0.83) [a]
Fact	5.80(1.01) [b]	5.43(1.02) [b]	6.14(0.81) [b]
No Rationale	3.15(1.51) [c]	2.48(1.51) [c]	4.63(1.60) [c]

Note: means without a superscript letter in common are significantly different from one another at $p < .05$.

Table S45
Mean ratings of vignettes across each condition for pro-gun restriction vignettes in pre-test

Vignette	Specificity	Evocative	Salient
Experience	5.50(1.13) [a]	4.92(1.30) [a]	5.77(0.95) [a]
Fact	4.73(1.16) [a]	5.19(1.28) [a]	6.04(0.85) [b]
No Rationale	3.33(1.43) [b]	2.60(1.35) [b]	4.97(1.26) [c]

Note: means without a superscript letter in common are significantly different from one another at $p < .05$.

Study 14: Replication in New Domain with Minority Opponents

Table S46

Mean Scores for manipulation check measures and dependent variables of Study 14.

	Facts Means(SD)	Personal Experience Means(SD)	No Rationale Means(SD)	Inferential
Personal Experience	4.21(1.35)[a]	6.04(0.98)[b]	4.47(1.06)[c]	$F(2,1894) = 477.83$, $p < .001$
Facts	5.03(1.27)[a]	3.08(1.37)[b]	3.76(1.18)[c]	$F(2,1894) = 377.28$, $p < .001$
Rational	4.66(1.48)[a]	5.05(1.42)[b]	3.84(1.40)[c]	$F(2,1894) = 117.07$, $p < .001$
Respect	5.05(1.25) [a]	5.40(1.15)[b]	4.70(1.33)[c]	$F(2,1892) = 50.16$, $p < .001$
Willingness to Interact	5.80(1.19) [a]	5.84(1.12)[a]	5.62(1.26)[b]	$F(2,1894) = 6.42$, $p = .002$

Note: means without a superscript letter in common are significantly different from one another at $p < .05$.

Participant ethnicity: Unfortunately, due to IRB regulations, we were unable to collect racial/ethnic information about participants in other studies. However, we received additional approval for these items for Study 14. The breakdown of participant ethnicity is as follows:

Ethnicity: N (% of sample)
White: 1410 (74.30%)
Black: 176 (9.3%)
American Indian or Alaska Native: 8 (0.4%)
Asian: 132 (7.0%)
Native Hawaiian or Pacific Islander: 4 (0.2%)
Hispanic or Latino: 54 (2.8%)
Biracial or Mixed Race: 97 (5.1%)
Other: 13 (0.7%)

Table S46

PROCESS Macro mediational analyses in Study 14

Outcome Variable	Mediator(s)	Indirect effect of Mediator(s) (SE), [95% CI]	Effect of Condition on Mediator (SE), [95% CI]	Effect of M1 on M2 (SE), [95% CI]	Effect of Mediator on DV (SE), [95% CI]	Total Effect (SE), [95% CI]	Direct Effect (SE), [95% CI]
	**Comparing Experiences (0) to Facts (1)**						
Respect	Rationality	B=-.23(.05) [-.23, -.02]	B=-.40(.08) [-.56, -.24]		B=.57(.02) [.54, .60]	B=-.35(.07) [-.49, -.21]	B=-.12(.05) [-.23, -.02]
Interact	Rationality (M1) Respect (M2)	B=-.11(.02) [-.16, -.07]	B=-.40(.08) [-.56, -.24]	B=.57(.02) [.54, .60]	B=.48(.03) [.43, .53]	B=-.05 (.07), [-.18, .08]	B=.12(.06), [.01, .23]
	**Comparing Experiences (0) to Control Condition (1)**						
Respect	Rationality	B=-.69(.05) [-.79, -.59]	B=-1.21(.08) [-1.37, -1.05]		B=.57(.02) [.54, .60]	B=-.70(.07) [-.84, -.56]	B=-.01(.06) [-.12, .1ß]
Interact	Rationality (M1) Respect (M2)	B=-.33(.03) [-.40, -.26]	B=-1.21(.08) [-1.37, -1.05]	B=.57(.02) [.54, .60]	B=.48(.03) [.43, .53]	B=-.23(.07) [-.36, -.10]	B=.12(.06) [-.004, .24]

Interaction between participant ethnicity and condition: The interaction term between participant ethnicity and condition on evaluations of rationality *F*(13, 1871)=1.51, p=0.11, respect *F*(13, 1869)=0.83, p=0.63, and willingness to interact *F*(13, 1871)=1.45, p=0.13 were non-significant.

Interaction between participant ideology and condition: There was a significant interaction between participant stance on guns and condition on evaluations of rationality of the opponent, *F*(10, 1879)=2.46, *p*=3.81. In general, people who were pro-restriction were viewed opponents as more rational (*M*=4.82, *SD*=1.32), as compared to pro- gun rights participants (*M*=3.91, *SD*=1.52). There was also significant interaction term between stances on gun policy and condition on ratings of respect, *F*(10, 1877)=3.42, *p*<.001. In general, people who were pro-restriction were viewed opponents as more rational (*M*=5.25, *SD*=1.14), as compared to pro- gun rights participants (*M*=4.71, *SD*=1.20).

Study 15: Perceptions of Scientists

Table S47

Mean Scores for manipulation check measures and dependent variables of Study 15.

	Facts Means(SD)	Experience Means(SD)	Scientist Means(SD)	Inferential
Personal Experience	2.93(1.44)[a]	5.75(1.14)[b]	3.94(1.30)[c]	$F(2,1407)=577.71, p<.001$
Facts	4.12(1.39)[a]	2.73(1.40)[b]	5.05(1.23)[c]	$F(2,1409)=350.49, p<.001$
Doubt	3.75(1.45)[a]	4.77(1.44)[b]	4.47(1.36)[c]	$F(2,1409)=65.07, p<.001$
Rational	3.61(1.62)[a]	4.02(1.71)[b]	4.20(1.54)[b]	$F(2,1408)=16.05, p<.001$
Respect	4.04(1.47)[a]	4.64(1.39)[b]	4.46(1.48)[c]	$F(2,1409)=21.78, p<.001$
Willingness to Interact	5.20(1.52)[a]	5.49(1.31)[b]	5.56(1.34)[b]	$F(2,1409)=8.52, p<.001$

Note: means without a superscript letter in common are significantly different from one another at $p < .05$. Higher scores on doubt measure indicate less doubt (i.e., greater perceived truth)

Interactions between participant ideology and condition: There was a significant interaction between participant stances on immigration and condition on ratings of opponent rationality, $F(2, 1405)=5.25$, $p=.01$. In general, participants who were pro-immigration viewed the opponent as less rational ($M=3.81$, $SD=1.60$), as compared to participants who were anti-immigration ($M=4.25$, $SD=1.70$). There was a non-significant interaction between participant stances on immigration and condition on ratings of respect towards the opponent, $F(2, 1406)=1.72$, $p=.18$—suggesting there were not differential effects of condition on ratings of respect based on participants stances on the political topic.

Table S48

PROCESS Macro mediational analyses in Study 15.

Outcome Variable	Mediator(s)	Indirect effect of Mediator(s) (SE), [95% CI]	Effect of Condition on Mediator (SE), [95% CI]	Effect of M1 on M2 (SE), [95% CI]	Effect of Mediator on DV (SE), [95% CI]	Total Effect (SE), [95% CI]	Direct Effect (SE), [95% CI]
			**Comparing Experiences to Facts**				
Rationality	Doubt	B=-.96(.09) [-1.13, -.78]	B=-1.02(.09) [-1.21, -.84]		B=.93(.02) [.90, .97]	B=-.41(.11) [-.62, -.20]	B=.54(.06) [.42, .68]
Respect	Doubt(M1) Rationality (M2)	B=-.38(.05) [-.48, -.29]	B=-1.02(.09) [-1.20, -.84]	B=.93(.02) [.90, .97]	B=.40(.03) [.34, .45]	B=-.61(.09) [-.79, -.42]	B=-.12(.07) [-.26, .01]
Respect	Rationality	B=-.26(.07) [-.39, -.12]	B=-.41(.11) [-.62, -.20]		B=.62(.02) [.59, .65]	B=-.61(.09) [-.79, -.42]	B=-.36(.07) [-.49, -.22]
Interact	Rationality (M1) Respect (M2)	B=-.13(.03) [-.20, .06]	B=-.41(.11) [-.62, -.20]	B=.62(.02) [.59, .65]	B=.48(.03) [.43, .55]	B=-.28(.09) [-.46, -.10]	B=.02(.08), [-.14, .17]
			**Comparing Experiences to Scientists**				
Rationality	Doubt	B=-.28(.09) [-.45, -.11]	B=-.30(.09) [-.48, -.12]		B=.93(.02) [.90, .97]	B=.18(.11) [-.03, .38]	B=.45(.06) [.33, .58]
Respect	Doubt(M1) Rationality (M2)	B=-.11(.04) [-.18, -.04]	B=-.30(.09) [-.48, -.12]	B=.93(.02) [.90, .97]	B=.40(.07) [.34, .45]	B=-.19(.09) [-.37, -.01]	B=-.17(.07) [-.30, -.04]
Respect	Rationality	B=.11(.07) [-.03, .24]	B=.17(.11) [-.03, .38]		B=.62(.02) [.59, .65]	B=-.19(.09) [-.37, -.01]	B=-.30(.07) [-.43, -.17]
Interact	Rationality (M1) Respect (M2)	B=.05(.03) [-.01, .12]	B=.18(.11) [-.03, .38]	B=.62(.02) [.59, .65]	B=.49(.03) [.43, .55]	B=.08(.09) [-.10, .25]	B=.17(.08) [.01, .32]

Note: First set of mediations, facts are the reference group. In the second set of mediations, the scientist condition is the reference group

Table S49
PROCESS Macro moderated mediation analyses with mean-centered Belief in Science Scale (BSS) as a moderator in Study 15[15].

Effect	Outcome Variable	Mediator	1 SD below mean BSS (SE), [95% CI]	Mean BSS (SE), [95% CI]	1 SD above mean BSS (SE), [95% CI]	Index of moderated mediation (SE), [95% CI]
Direct	Rationality	Doubt	B=.34(.08) [.18, .51]	B=.45(.06) [.33, .57]	B=.58(.09) [.41, .75]	.14(.05) [.04, .23]
Indirect	Rationality	Doubt	B=-.52(.12) [-.76, -.27]	B=-.29(.09) [-.46, -.13]	B=-.02(.12) [-.27, .22]	
Direct	Respect	Doubt(M1) Rationality (M2)	B=-.26(.09) [-.44, -.08]	B=-.18(.07) [-.31, -.05]	B=-.08(.09) [-.27, .10]	.05(.02) [.02, .09]
Indirect	Respect	Doubt(M1) Rationality (M2)	B=-.21(.05) [-.31, -.11]	B=-.12(.04) [-.19, -.05]	B=-.009(.05) [-.11, .09]	
Direct	Respect	Rationality	B=-.39(.09) [-.57, -.21]	B=-.31(.07) [-.44, -.18]	B=-.21(.10) [-.40, -.03]	.12(.04) [.05, .20]
Indirect	Respect	Rationality	B=-.11(.09) [-.29, .08]	B=.10(.06) [-.03, .23]	B=.35(.09) [.16, .53]	
Direct	Interact	Rationality (M1) Respect (M2)	B=.11(.11) [-.10, .32]	B=.16(.08) [.007, .32]	B=.22(.08) [.007, .32]	.06(.02) [.02, .10]
Indirect	Interact	Rationality (M1) Respect (M2)	B=-.05(.05) [-.15, .04]	B=.05(.03) [-.02, .11]	B=.17(.05) [.08, .26]	

Note: These mediations are comparing the experiential condition to the scientist condition (reference group). Models with 1 mediator were run using model 8, models with 2 mediators were run using model 85.

[15] Currently PROCESS macro does not support testing moderation with a two-mediator mediational pathway. Therefore, we were unable to test whether BSS moderated the model between condition and willingness to interact with the two mediators (rationality and subsequent respect).

Based on Table S52 we see in general, effects are dependent on the level at which individuals believe in science. The more people believe in science, the less there are differences between perceptions of experiential and scientific targets.

Table S50

PROCESS Macro moderated mediation analyses with mean-centered participant political ideology (PPI) as a moderator in Study 15.

Effect	Outcome Variable	Mediator	1 SD below mean PPI (SE), [95% CI]	Mean PPI (SE), [95% CI]	1 SD above mean PPI (SE), [95% CI]	Index of moderated mediation (SE), [95% CI]
Direct	Rationality	Doubt	B=.47(.08) [.32, .62]	B=.45(.06) [.33, .58]	B=.42(.08) [.26, .57]	-.09(.05) [-.18, .003]
Indirect	Rationality	Doubt	B=-.17(.10) [-.38, .03]	B=-.26(.08) [-.42, -.09]	B=-.43(.11) [-.65, -.21]	
Direct	Respect	Doubt(M1) Rationality (M2)	B=-.25(.09) [-.41, -.09]	B=-.20(.07) [-.34, -.07]	B=-.11(.08) [-.27, .06]	-.03(.02) [-.07, .001]
Indirect	Respect	Doubt(M1) Rationality (M2)	B=-.07(.04) [-.15, .02]	B=-.10(.03) [-.17, -.03]	B=-.009(.05) [-.26, -.08]	
Direct	Respect	Rationality	B=-.37(.08) [-.54, -.21]	B=-.33(.07) [-.47, -.20]	B=-.25(.08) [-.41, -.08]	-.06(.03) [-.13, .004]
Indirect	Respect	Rationality	B=.18(.07) [.03, .34]	B=.11(.06) [-.01, .25]	B=-.01(.08) [-.17, .16]	
Direct	Interact	Rationality (M1) Respect(M2)	B=.13(.10) [-.06, .33]	B=.16(.08) [.002, .32]	B=.22(.10) [.02, .41]	-.03(.02) [-.07, .001]
Indirect	Interact	Rationality (M1) Respect (M2)	B=.09(.04) [.02, .17]	B=.06(.03) [-.004, .12]	B=-.004(.04) [-.09, .08]	

Note: These mediations are comparing the experiential condition to the scientist condition (reference group). Higher scores on PPI = greater conservatism. Models with 1 mediator were run using model 8, models with 2 mediators were run using model 85.

Links for Pre-Registrations

Study 1: Lay Belief- Free Response	https://aspredicted.org/blind.php?x=fk97mb
Study 3: YouTube Comments.	https://osf.io/j4czu/.
Study 4: Testing the Model	https://aspredicted.org/blind.php?x=yr3a4b
Study 5: Field Study	http://aspredicted.org/blind.php?x=b652d7
Study 6: Specific Facts	https://aspredicted.org/blind.php?x=nm7xc4
Study 7: Non-Relevant Experiences.	https://aspredicted.org/blind.php?x=je6yb4
Study 8: Non-Harmful Experiences	https://aspredicted.org/blind.php?x=tb6zi2
Study 9: Experience Gradient- Pilot Study	https://aspredicted.org/blind.php?x=3sm9sh
Study 9: Experience Gradient	https://aspredicted.org/blind.php?x=e9t8hz
Study 10: Real Experiences vs. Real Facts	https://aspredicted.org/blind.php?x=mw6tc7
Study 11: Real Op-ed Articles- Pilot Study	https://aspredicted.org/blind.php?x=zh5ek4
Study 11: Real Op-ed Articles	https://aspredicted.org/blind.php?x=bi64qg
Study 12: News Transcripts	https://aspredicted.org/blind.php?x=qw8u43
Study 13: Doubt and Moral vs Non-Moral Contexts	https://aspredicted.org/blind.php?x=w4p6j5
Study 14: Replication with Black Female Opponents: Pre-Test	https://aspredicted.org/blind.php?x=tn4bn5
Study 14: Replication with Black Female Opponents:	https://aspredicted.org/blind.php?x=t4355a
Study 15: Perceptions of Scientists	https://aspredicted.org/blind.php?x=my9rb4

Additional Study: Replication in New Domain (Black Lives Matter vs Blue Lives Matter)

We conducted an additional study but decided to not include it in the main manuscript as there were several concerns about the interpretation of effects. In this study[16](N=771)—we sought to generalize our results to a new issue (race and policing) and examine whether the mere *potential* for future harm is sufficient for fostering perceived rationality and therefore respect. This study also included a strict control condition in which opponents simply asserted their political viewpoint without mentioning facts or personal experiences. This allowed us to reveal whether facts were at least better than nothing in political disagreements. In the previous studies, the opponents who based their stance on facts also explicitly mentioned the source of those facts (e.g., read in a governmental report), which could have seemed awkward and made the fact seem less credible. We revised the vignettes in this study to not include such language.

In this study, participants first reported their stance on race and policing by indicating whether they supported the Black Lives Matter (BlackLM) movement or the Blue Lives Matter (BlueLM) movement. Participants read about an opponent who disagreed with them, split by four conditions: 1) a control condition where an Asian woman provides nothing to justify her beliefs, 2) a facts condition[17] where an Asian woman cites statistics about the dangerousness of police encounters for Black men (BlackLM) or police officers (BlueLM), 3) a close-other experience condition where an Asian woman mentions that her adopted son is Black (BlackLM) or a police officer (BlueLM) and is worried about his safety, 4) a personal experience condition where a Black man is worried about being killed by police (BlackLM) or where a Black woman is a police officer and is worried about being killed on the job (BlueLM).

We selected these targets intentionally, wanting to use the experiences of Black Americans in the personal experience condition but not in other conditions. The nature of the issue means that participants would likely infer some relevant harm-based personal experience of any Black American target, even in the facts or control condition. We conducted a comprehensive pilot study that confirmed the equivalence of the facts and personal experiences on a variety of potential confounds including convincingness, harm, specificity, evocativeness, and salience. In all but one case,[18] ratings were either equivalent or factual vignettes were rated as higher (see supporting information).

Analyses revealed a main effect of condition on perceived rationality $F(3,767)=18.88$, $p<.001$, and respect $F(3, 765)=11.30$, $p<.001$. Post-hoc analyses indicated that, compared with both the control condition (Rationality: M=3.57, SD=1.56; Respect: M=4.28, SD=1.62) and the facts condition (Rationality: M=4.16, SD=1.61; Respect: M=4.59, SD=1.62) condition, the personal experience condition was significantly higher on both perceived rationality (M=4.77, SD=1.55) and respect (M=5.14, SD=1.45), (all comparisons significant at $p< .001$), see Figure 6. Mediation analyses revealed that personal experiences (vs. facts) fostered respect through

[16] The pretest of this study was registered here: http://aspredicted.org/blind.php?x=7zn3kv, and the full study was pre-registered here: https://aspredicted.org/blind.php?x=8hr7je

[17] Of note, the facts we included highlighted real and obvious harm (i.e., killings of African Americans and police officers).

[18] In the pre-test of the Blue Lives Matter vignettes, the self-experiential vignette was rated as significantly more convincing ($p<.001$). We believe this is due to a violation of base-rates, with the target being a Black woman who supports the Blue Lives Matter movement. On all other dimensions these vignettes were either comparable, or the fact vignette was rated higher.

rationality, indirect effect=-.45, SE=.12, 95% CI [-.69, -.21]. The total effect of the model was significant as well *b*=-.56, SE=.16, 95% CI [-.87, -.26].

These results replicate key findings from our past studies: personal, harm-based experiences foster respect more than facts. This study reveals that the power for personal experiences to bridge moral/political divides generalizes to the experiences of people of color and to the *potential* for future harm. The study also found that facts, even if less respect-fostering than personal experiences, are better than providing no justification at all.

Pre-Test: To pre-test the items we recruited 194 participants from MTurk. Participants read a subset of the 8 vignettes, they either read all the Blue Lives Matter vignettes, or all the Black Lives Matter vignettes. Which set they read was randomized and not based on whether they supported either movement. We tested two forms of personal experience vignettes, one highlighted concern the opponent themselves could personally experience a potential harm, the other highlighted concerns the opponent had that a close other could experience a potential harm. The fact condition discussed real harm surrounding racism and policing, and the no rationale condition acted as a control condition in which the opponent did not provide a reason for holding their stance. The vignettes can be found in Table S51. All variables on all vignettes had acceptable to high reliability (minimum Cronbach α = .74).

After reading each vignette (in a randomized order), participants reported the extent to which each vignette highlighted several dimensions (i.e., how convincing, harmful, specific, evocative, and salient each vignette was). All dimensions used a 7-point scale from *strongly disagree* (1) to *strongly agree* (7). The items used for each dimension can be found in Table S52.

Analyses indicated personal experience and factual vignettes were overall comparable. In most cases there were no significant differences between these vignettes, and when there were, the factual vignette was rated higher, which in theory "stacks the cards" further against our favor (i.e., making it less like personal experiences would be rated as more rational or incite more respt). However, we would like to note that in one unique circumstance; the highly experiential Blue Lives Matter vignette was rated as more convincing than the factual Blue Lives Matter vignette. We believe this could be due to the fact that the target (a black woman) who not only was a police officer, but supported the Blue Lives Matter movement. See Table S53 and S54 for mean ratings for each vignette across dimensions.

Table S51

Vignettes used in additional study

Condition	Vignette
Low Personal Experience	Paula Zhao is an Asian American who supports the [Black/Blue] Lives Matter movement. When she talks to people about it, she tells them that it's because her adopted son is [Black/a police officer] and she worries about him being killed [by the police/on the job.
High Personal Experience	[Tyron/Tia] Williams is a Black American who supports the [Black/Blue] Lives Matter movement. When [he/she] talks to people about it, [he/she] tells them that it's because, as a [black man/police officer], [he/she] worries about being killed [at the hands of police/while on the job].
Fact	Samantha Lee is an Asian American who supports the [Black/Blue] Lives Matter movement. When she talks to people about it, she tells them that it's because [African Americans account for less than 13% of the U.S. population but are killed by police officers at twice the rate of White Americans[19]/ there was a 13% increase in the number of police officers killed on the job since 2017][20].
No Rationale	Sarah Zhang is an Asian American who supports the [Black/Blue] Lives Matter movement.

[19] This statistic came from the following source: https://www.washingtonpost.com/graphics/investigations/police-shootings-database/

[20] This statistic came from the following source: https://www.forbes.com/sites/niallmccarthy/2019/05/08/the-number-of-u-s-police-officers-killed-in-the-line-of-duty-increased-last-year-infographic/#4d8c90af1189

Table S52

Variables and reliability of items used to assess comparability of vignettes in additional study pre-test

Variable	Items
Convincing	1. "This passage was powerful."<br>2."The passage seemed compelling."<br>3."I found the passage impactful."
Harm	1."The passage involves suffering."<br>2."The passage highlights victimization."<br>3."This passage focuses on harm."
Specificity	1."The statement is… concrete"<br>2." The statement is… abstract"<br>3." The statement is… vague"<br>4." The statement is… specific"<br>5." The statement is… detailed"
Evocative	1." The statement … is detailed and descriptive"<br>2." The statement… captures my attention"<br>3." The statement … has vivid language"
Salient	1." The statement is … related to an important discussion<br>in the United States"<br>2." The statement is… relevant to society"<br>3." The statement is … meaningful for our understanding<br>of social and political issues in this country "

Table S53

Mean ratings of vignettes across each condition for Black Lives Matter vignettes in pre-test

Vignette	Convince	Harm	Specificity	Evocative	Salient
Low Experience	4.64(1.48) [ab]	4.69(1.27) [a]	4.84(1.21) [a]	4.29(1.34) [a]	5.44(1.11) [a]
High Experience	4.89(1.57) [a]	4.85(1.33) [ab]	4.92(1.03) [ab]	4.39(1.38) [a]	5.67(1.14) [a]
Fact	4.31(1.66) [b]	5.08(1.09) [b]	5.23(1.11) [b]	4.48(1.34) [a]	5.60(.99) [a]
No Rationale	2.42(1.58) [c]	2.16(1.42) [c]	2.97(1.36) [c]	2.18(1.34) [b]	4.30(1.57) [b]

Note: means without a superscript letter in common are significantly different from one another at $p < .05$.

Table S54

Mean ratings of vignettes across each condition for Blue Lives Matter vignettes in pre-test

Vignette	Convince	Harm	Specificity	Evocative	Salient
Low Experience	5.01(1.66) [a]	5.26(1.21) [a]	5.01(1.21) [a]	4.43(1.45) [a]	5.79(1.40) [a]
High Experience	5.04(1.62) [a]	5.62(1.11) [b]	4.86(1.27) [a]	4.48(1.51) [a]	5.79(1.40) [a]
Fact	5.11(1.57) [a]	5.75(.96) [b]	5.53(1.04) [b]	5.12(1.18) [b]	5.86(1.32) [a]
No Rationale	2.91(1.70) [b]	2.46(1.57) [c]	3.30(1.41) [c]	2.58(1.64) [c]	4.55(1.70) [b]

Note: means without a superscript letter in common are significantly different from one another at $p < .05$.

Full Study: For the full study, participants reported their stance on policing in the United States by choosing which option fits their beliefs the best using a 6-point scale from, *I completely support the Black Lives Matter movement* (1) to *I completely support the Blue Lives Matter movement* (7). Participants were then randomly assigned to read about an opponent who disagreed with them based on protentional harms of a close other, personal experience (i.e., worries they themselves would experience harm), facts (about actual harm), or they did not provide a rationale (control condition). Participants then responded to our standard manipulation check and rationality, respect, interact measures.

Table S55

ANOVA results for each study variable by condition

Vignette	Personal Experience M(SD)	Facts M(SD)	Rational M(SD)	Respect M(SD)	Interact M(SD)
Potential Harm for Close Other	5.08(1.21) [a]	3.06(1.42) [a]	4.33(1.66) [a]	4.83(1.35) [a]	5.62(1.28) [a]
Potential Harm for Self	5.68(1.34) [b]	3.84(1.43) [b]	4.77(1.55) [b]	5.14(1.45) [b]	5.75(1.30) [a]
Fact	3.38(1.34) [c]	4.56(1.33) [c]	4.16(1.61) [a]	4.59(1.60) [a]	5.58(1.17) [a]
Control	3.72(1.24) [d]	3.71(1.29) [b]	3.57(1.56) [c]	4.28(1.62) [c]	5.26(1.56) [b]
Statistic	$F(3,767)=139.48$, $p<.001$	$F(3,767)=38.88$, $p<.001$	$F(3,765)=18.88$, $p<.001$	$F(3,765)=11.30$, $p<.001$	$F(3,767)=4.76$, $p=.003$

Note: means without a superscript letter in common are significantly different from one another at $p < .05$[21].

[21] In our pre-registration we predicted that there would not be significant differences between our two versions of the experiential condition. However, in our full study it seems people viewed concerns about oneself being harmed as having significantly more personal experience, this in turn seems to have shaped the downstream consequences of rationality and willingness to respect.

Table S56

PROCESS mediational Analysis Results for additional study

Outcome Variable	Mediator(s)	Indirect effect of Mediator(s) (SE), [95% CI]	Effect of Condition on Mediator (SE), [95% CI]	Effect of M1 on M2 (SE), [95% CI]	Effect of Mediator on DV (SE), [95% CI]	Total Effect (SE), [95% CI]	Direct Effect (SE), [95% CI]
		**Comparing Facts (1) vs Own PE (0)**					
Respect	Rationality	B=-.45(.12), [-.69, -.21]	B=-.62(.16), [-.94, -.30]		B=.73(.02), [.69, .77]	B=-.56(.15), [-.87, -.26]	B=-.12(.10), [-.31, .08]
Interact	Rationality (M1) Respect (M2)	B=-.21(.06), [-.32, -.10]	B=-.62(.16), [-.94, -.30]	B=.73(.02), [.69, .77]	B=.47(.04), [.39, .55]	B=-.15(.14), [-.42, .11]	B=.14(.11), [-.09, .36]
		**Own PE (1) vs No Rationale (0)**					
Respect	Rationality	B=.88(.12), [.65, 1.11]	B=1.20(.16), [.88, 1.52]		B=.73(.02), [.69, .77]	B=.86(.15), [.56, 1.16]	B=-.02(.10), [-.22, .18]
Interact	Rationality (M1) Respect (M2)	B=.41(.07), [.28, .56]	B=1.20(.16), [.88, 1.52]	B=.73(.02), [.69, .77]	B=.47(.04), [.39, .55]	B=.49(.14), [.22, .76]	B=.04(.12), [-.19, .27]
		**Own PE (1) vs Adopted Son PE (0)**					
Respect	Rationality	B=.33(.12), [.09, .56]	B=.45(.16), [.12, .77]		B=.73(.02), [.69, .77]	B=.31(.15), [.004, .61]	B=-.02(.10), [-.22, .18]
Interact	Rationality (M1) Respect (M2)	B=.15(.06), [.04, .27]	B=.45(.16), [.12, .77]	B=.73(.02), [.69, .77]	B=.47(.04), [.39, .55]	B=.13(.14), [-.14, .40]	B=-.03(.11), [-.26, .19]
		**Facts (1) vs Adopted Son PE (0)**					
Respect	Rationality	B=-.12(.12), [-.37, .12]	B=-.17(.16), [-.49, .15]		B=.73(.02), [.69, .77]	B=-.23(.16), [-.56, .05]	B=-.13(.10), [-.33, .06]
Interact	Rationality (M1) Respect (M2)	B=-.05(.06), [-.17, .05]	B=-.17(.16), [-.49, .15]	B=.73(.02), [.69, .77]	B=.47(.04), [.39, .55]]	B=-.02(.14), [-.29, .25]	B=.10(.11), [-.12, .33]
		**Facts (1) vs No Rationale (0)**					
Respect	Rationality	B=.43(.12), [.19, .66]	B=-.17(.16), [.27 .91]		B=.73(.02), [.69, .77]	B=-.29(.15), [-.007, .60]	B=-.13(.10), [-.33, .06]
Interact	Rationality (M1) Respect (M2)	B=.20(.06), [.08, .33]	B=.59(.16), [.27, .91]	B=.73(.02), [.69, .77]	B=.47(.04), [.39, .55]	B=.34(.14), [.07, .61]	B=.17(.11), [-.05, .40]

Note: (1) denotes that condition was the reference group in the model, (0) means it was the comparison group.

Interaction between participant ideology and condition: There was a non-significant but trending interaction term between participant stance on policing and condition on evaluations of rationality of the opponent, $F(3, 761)=2.27$, $p=.08$. In general, pro- Black Lives Matter participants rated the opponent as more rational ($M=4.36$, $SD=1.54$), as compared to pro- Blue Lives Matter participants ($M=3.91$, $SD=1.82$). There was not a significant interaction term between stances on gun policy and condition on ratings of respect, $F(3, 761)=.71$, $p=.54$.

References for Supporting Information

1. J. W., Pennebaker, R. J. Booth, R. L. Boyd, M. E. Francis, *Linguistic inquiry and word count: LIWC2015*. Austin, TX: Pennebaker Conglomerates (2015).
2. A. Aron, E. N. Aron, D. Smollan, Inclusion of other in the self-scale and the structure of interpersonal closeness. *Journal of Personality and Social Psychology, 63*(4). 596-612 (1992).
3. G. S. Morgan, L. J. Skitka, D. C. Wisneski, Moral and religious convictions and intentions to vote in the 2008 presidential election. *Analyses of Social Issues and Public Policy, 10*(1), 307-320 (2010).
4. M. Farias, A. Newheiser, G. Kahane, Z. de Toledo, Scientific faith: Belief in science increases in the face of stress and existential anxiety. *Journal of Experimental Social Psychology, 49*(6), 1210-1213 (2013).
5. S. L. Thomas, R. H. Heck, K. W. Bauer, Weighting and adjusting for design effects in secondary data analyses. *New Directions for Institutional Research, 127,* 51-72 (2005).
6. K. J. Preacher, M. J. Zyphur, Z. Zhang, A general multilevel SEM framework for assessing multilevel mediation. *Psychological Methods, 15*(3), 209 (2010).
7. D. L. Oberski, Evaluating sensitivity of parameters of interest to measurement invariance in latent variable models. *Political Analysis,* 45-60 (2014).
8. A. F. Hayes, N. J. Rockwood, Conditional process analysis: Concepts, computation, and advances in the modeling of the contingencies of mechanisms. *American Behavioral Scientist, 64*(1), 19-54 (2020).
9. K. J. Preacher, M. J. Zyphur, Z. Zhang, A general multilevel SEM framework for assessing multilevel mediation. *Psychological Methods, 15*(3), 209-233 (2010).
